# Apolipoprotein E genotype does not moderate the associations of depressive symptoms, neuroticism and allostatic load with cognitive ability and cognitive aging in the Lothian Birth Cohort 1936

**DOI:** 10.1371/journal.pone.0192604

**Published:** 2018-02-16

**Authors:** Zander Crook, Tom Booth, Simon R. Cox, Janie Corley, Dominika Dykiert, Paul Redmond, Alison Pattie, Adele M. Taylor, Sarah E. Harris, John M. Starr, Ian J. Deary

**Affiliations:** 1 Department of Psychology, The University of Edinburgh, Edinburgh, United Kingdom; 2 Centre for Cognitive Ageing and Cognitive Epidemiology, Department of Psychology, The University of Edinburgh, Edinburgh, United Kingdom; 3 Medical Genetics Section, Centre for Genomic and Experimental Medicine, Medical Research Council Institute of Genetics and Molecular Medicine, The University of Edinburgh, Edinburgh, United Kingdom; 4 Alzheimer Scotland Dementia Research Centre, Department of Psychology, The University of Edinburgh, Edinburgh, United Kingdom; Duke University, UNITED STATES

## Abstract

**Objectives:**

In this replication-and-extension study, we tested whether depressive symptoms, neuroticism, and allostatic load (multisystem physiological dysregulation) were related to lower baseline cognitive ability and greater subsequent cognitive decline in older adults, and whether these relationships were moderated by the E4 allele of the apolipoprotein E (*APOE*) gene. We also tested whether allostatic load mediated the relationships between neuroticism and cognitive outcomes.

**Methods:**

We used data from the Lothian Birth Cohort 1936 (*n* at Waves 1–3: 1,028 [*M* age = 69.5 y]; 820 [*M* duration since Wave 1 = 2.98 y]; 659 [*M* duration since Wave 1 = 6.74 y]). We fitted latent growth curve models of general cognitive ability (modeled using five cognitive tests) with groups of *APOE* E4 non-carriers and carriers. In separate models, depressive symptoms, neuroticism, and allostatic load predicted baseline cognitive ability and subsequent cognitive decline. In addition, models tested whether allostatic load mediated relationships between neuroticism and cognitive outcomes.

**Results:**

Baseline cognitive ability had small-to-moderate negative associations with depressive symptoms (*β* range = -0.20 to -0.17), neuroticism (*β* range = -0.27 to -0.23), and allostatic load (*β* range = -0.11 to 0.09). Greater cognitive decline was linked to baseline allostatic load (*β* range = -0.98 to -0.83) and depressive symptoms (*β* range = -1.00 to -0.88). However, *APOE* E4 allele possession did not moderate the relationships of depressive symptoms, neuroticism and allostatic load with cognitive ability and cognitive decline. Additionally, the associations of neuroticism with cognitive ability and cognitive decline were not mediated through allostatic load.

**Conclusions:**

Our results suggest that *APOE* E4 status does not moderate the relationships of depressive symptoms, neuroticism, and allostatic load with cognitive ability and cognitive decline in healthy older adults. The most notable positive finding in the current research was the strong association between allostatic load and cognitive decline.

## Introduction

Cognitive aging is an increasingly important public health issue [1]. It is therefore crucial that we aim to improve our understanding of the risk factors for negative cognitive outcomes in old age and how these risk factors interact, so that we can work towards accurately identifying and optimally treating those at higher risk of harmful cognitive aging.

Three such risk factors are the E4 allele of the apolipoprotein E (*APOE*) gene, depression and neuroticism. People with major depressive disorder tend to have higher than average neuroticism [2], so the relationships between neuroticism and cognitive outcomes will tend to overlap with the relationships between depression and cognitive outcomes. Studying both sets of relationships is valuable, because neuroticism is a broader construct than depression. It comprises several facets, including anxiety, hostility, self-consciousness and vulnerability to stress [3].

### The relationships of *APOE* E4, depressive symptoms and neuroticism with cognitive ability

The *APOE* E4 allele, depression and neuroticism have each been found to have adverse associations with contemporaneously measured cognitive ability and subsequent cognitive decline. Possession of the E4 allele of the *APOE* gene has been linked to higher risk of late-onset Alzheimer’s disease [4] as well as lower cognitive ability [5] and greater cognitive decline [6] in non-clinical groups of older adults. Additionally, depression at a young age seems to increase risk for dementia, and in old age, depression may sometimes be prodromal of dementia [7]. In cognitively healthy individuals, depressive symptomatology and neuroticism have each been related to worse cognitive ability and greater cognitive decline [8–10]. Also, *APOE* genotype has been linked to the variability of depressive symptomatology and spatial reasoning within pairs of monozygotic twins [11]. This result suggested that for those outcomes, *APOE* genotype may moderate the effects of some environmental variables.

### *APOE* E4 moderation of the relationships depressive symptoms and neuroticism have with cognitive ability

Recent research has investigated whether *APOE* genotype moderates the associations of depression and neuroticism with cognitive ability and decline such that they are stronger in E4 allele carriers. If these moderating effects were confirmed, this would have implications for the treatment of older depressed and neurotic people and help us to understand some of the mechanisms underlying these relationships. Longitudinal studies have found that depression increases dementia and mild cognitive impairment risk more in E4 carriers than in E4 non-carriers [12–16]. Plus, studies using cognitive impairment screeners to measure cognitive ability have reported that depressive symptoms have a stronger association with cognitive ability and decline in E4 allele carriers [17, 18]. However, cognitive impairment screening tests are known to exhibit ceiling effects when administered in non-impaired populations [19, 20]. As such, to test the moderation by E4 status (carrying vs. not carrying at least one E4 allele) in relation to the full range of healthy cognitive ability, it is preferable to use finer-grained cognitive tests. Only one of three studies that used multiple, more challenging cognitive tests to measure cognition reported that depressive symptoms were more strongly related to cognition in E4 carriers ([21], cf. [22–24]). Regarding neuroticism, in the Ginkgo Evaluation of Memory sample neuroticism has a greater negative association with cognitive ability [25] and subsequent cognitive decline [26] in E4 carriers. In the Victoria Longitudinal Study sample, however, *APOE* E4 status did not moderate the relationships of neuroticism with baseline declarative memory and subsequent decline in declarative memory [24]. Furthermore, a cross-sectional study of 172 non-demented older adults based in Rochester, New York, USA, found that there was overall *APOE*-by-neuroticism interaction when predicting scores on five cognitive domains, although the only specific domain for which the interaction was statistically significant was attention [27].

### Viewing the *APOE*-by-neuroticism interaction within an allostatic load framework

Dar-Nimrod et al. put forward that the interaction between neuroticism and *APOE* E4 status can be thought of within "an allostatic load framework" ([25], p. 1148). Allostatic load (AL) refers to the accumulation of damage to the body from the dysregulation of physiological systems [28]. AL is typically operationalised by calculating summary scores for participants using biomarker data from multiple physiological systems, such as the metabolic, neuroendocrine, cardiovascular and immune systems [29]. Ideally, AL summary scores would be calculated using many biomarkers from a wide range of physiological systems, including both primary mediators of stress responses and secondary outcome biomarkers whose levels change in response to primary mediators’ levels. Most commonly, AL summary scores are sums of at-risk biomarkers, where risk is indicated by sample quantiles or clinical thresholds [30]. A comparative study found that scoring methods with higher criterion validity were those that retained continuous measurement of the biomarkers and/or incorporated risk at both high and low levels of biomarkers [31]. More recently, some researchers have generated scores through modeling AL as a latent factor that causes AL biomarker values [32, 33,], although this method has been called into question based on the assumptions underlying these models [34].

Specifically, Dar-Nimrod et al. [26] posited that this interaction may function through increased hypothalamic–pituitary–adrenal (HPA) axis activity, with "inflammatory and metabolic processes" ([25], p. 1152) also possibly playing a role. The hypothesis put forward by Dar-Nimrod et al. is directly testable using moderated-mediation models [35]. In such a model, the effect of neuroticism on cognitive ability and decline would be partially mediated through physiological dysregulation, and the second stage of this mediation process, from physiological dysregulation to cognition, would be moderated by *APOE* E4 status.

Some previous research reports findings consistent with Dar-Nimrod et al.’s [25, 26] supposition. Individuals with high neuroticism tend to display maladaptive HPA axis activity [36], have higher inflammation [37, 38,], and be at higher risk of metabolic syndrome [39]. A recent study found that higher neuroticism was related to higher AL, although higher AL did not predict change in neuroticism over a four-year period [40]. Studies of older adults have reported that higher cortisol levels more strongly predict decrements in contemporaneously measured cognitive ability [41] and subsequent cognitive decline [42] in *APOE* E4 carriers. Additionally, higher AL has been linked to greater depressive symptomatology [43] as well as worse cognitive ability [31, 44, 45] and subsequent decline [46–48].

### The present study

In the present study, we tested whether the effects of depressive symptoms, neuroticism and AL on baseline general cognitive ability and subsequent general cognitive decline were moderated by *APOE* E4 possession. Specifically, we tested the following sequence of research questions:

1. Do *APOE* E4 non-carriers and carriers differ in their baseline cognitive ability and in their levels of subsequent change in cognitive ability?
2. Are baseline depressive symptoms, neuroticism and allostatic load related to baseline cognitive ability and subsequent change in cognitive ability?
3. Are the relationships of baseline depressive symptoms, neuroticism and allostatic load with baseline cognitive ability and subsequent cognitive change moderated by *APOE* status?
4. Are the relationships of baseline neuroticism with baseline cognitive ability and subsequent cognitive change mediated by allostatic load?
5. Are these mediated effects moderated by *APOE* status?

Previous studies using this sample have tested some of the main effects of the present study, such as the associations of *APOE* genotype [49, 50], time [36, 51], depressive symptoms [52] and AL [45] with cognitive ability. However, this is the first study using this sample to test the *APOE* moderation of the relationships of depressive symptoms, neuroticism and allostatic load with cognitive outcomes, as well as the first to test the association of AL with change in cognitive ability during older age.

## Methods

### Participants

The sample used was the Lothian Birth Cohort 1936 (LBC1936). On June 4, 1947, almost all school children in Scotland born in 1936 took the Moray House Test No. 12, a validated intelligence test, as part of the Scottish Mental Survey 1947 [53]. The LBC1936 study recruited surviving Scottish Mental Survey 1947 participants living in the Lothian region (the City of Edinburgh and its surrounding area) to investigate cognitive aging. The LBC1936 study design, including the recruitment and testing of the sample, has been detailed in two cohort profiles [54, 55].

Briefly, data was used from the LBC1936 baseline wave, and follow-up waves approximately three and seven years post-baseline. Data was collected for LBC1936 Wave 1 in 2004–2007 (*N* = 1091 [543 females, 548 males]; *M* [*SD*] age = 69.5 [0.8] y), for Wave 2 in 2007–2010 (*n* = 866 [418 females, 448 males]; *M* [*SD*] age = 72.5 [0.7] y; *M* [*SD*] time since Wave 1 data collection = 2.98 [0.28] y), and for Wave 3 in 2011–2013 (*n* = 697 [337 females, 360 males]; *M* [*SD*] age = 76.3 [0.7] y; *M* [*SD*] time since Wave 1 data collection = 6.74 [0.31] y).

Ethical permission for the LBC1936 study protocol was obtained from the Multi-Centre Research Ethics Committee for Scotland (MREC/01/0/56) and from the Lothian Research Ethics Committee (LREC/2003/2/29). The research was carried out in compliance with the Helsinki Declaration. All subjects gave written, informed consent.

The number of participants with each genotype was: E2/E2, 5; E2/E3, 120; E2/E4, 23; E3/E3, 597; E3/E4, 262; E4/E4, 21. *APOE* in the current sample has previously been shown to be in Hardy-Weinberg equilibrium (*p* = 0.62) [49] All study participants were white. The number of participants who self-reported having dementia was 10 in total: two at Wave 2 and nine at Wave 3, which included one of the two at Wave 2.

### Measures

Cognitive test, age and medical condition data came from LBC1936 Waves 1, 2 and 3. Depressive symptoms, neuroticism and allostatic load data came from LBC1936 Wave 1.

### *APOE* genotyping

Participants’ DNA was isolated from whole blood samples. To determine which *APOE* alleles individuals possessed, the single nucleotide polymorphisms rs7412 and rs429358 were genotyped using TaqMan assays (Applied Biosystems, Carlsbad, CA, USA). This was done by the Wellcome Trust Clinical Research Facility Genetics Core at the Western General Hospital in Edinburgh.

### General cognitive ability

The full cognitive test battery administered to the participants was detailed in the LBC1936 cohort profile [54]. The present study used data from the following five subtests of the Wechsler Adult Intelligence Scale III^UK^ [56]: Digit Symbol Coding, a processing speed test where participants enter symbols based on a provided number-symbol code; Block Design, a reasoning test where participants must use blocks to duplicate a given design; Symbol Search, a processing speed test where participants must indicate whether rows of symbols include either of two target symbols; Letter-Number Sequencing, a working memory test that requires ordered recall of unordered numbers and letters; and Matrix Reasoning, where participants must select the correct piece to complete a patterned matrix. Collectively, these tests primarily assess higher-order executive functions, which mainly tap the frontal lobe.

#### Cognitive impairment screening

The Mini Mental State Examination (MMSE) [57] was used as a test of global cognitive status. It is a brief measure that was designed to identify cognitive impairment and assess its extent [57]. The MMSE has been part of the LBC1936 protocols since the outset as a screening test for dementia onset.

#### Depressive symptoms

The seven-item depression subscale of the Hospital Anxiety and Depression Scale (HADS-D) [58] was used to measure depressive symptoms. Item-level data was unavailable, so instead of depressive symptoms being modeled as a latent factor, observed subscale scores were used.

#### Neuroticism

The 10-item emotional stability subscale of a 50-item Big Five factor markers scale [59] from the International Personality Item Pool (IPIP) [60] was reverse scored to serve as a measure of neuroticism. We modelled neuroticism as a latent variable using item parcels, which are sums of different item responses [61]. We used item parcels to normalize the distributions of manifest variables and to facilitate model fitting by keeping the model compact, with as few variables as were required. Primary analyses used three item parcels, each of which contained items about different facets of neuroticism so that each parcel was representative of the broader domain of neuroticism. The parcels which were formed from three (items 4, 34, 49), four (9, 14, 29, 39) and three (19, 24, 44) items respectively. An individual’s score on each parcel was the mean score of the items they answered, or coded as missing if they had not answered half or more of the parcel’s items. Neuroticism was then modeled as a latent variable, the common cause of the variance shared by the item parcels. This enabled us to test the measurement invariance of neuroticism across groups (see Statistical Analysis section).

#### Allostatic load

AL scores were mean absolute *z* scores from nine biomarkers: albumin; C-reactive protein (CRP); fibrinogen; glycated haemoglobin (HbA1c); high-density lipoprotein ratio (HDL ratio or HDLR); triglycerides; body mass index (BMI); systolic blood pressure (SBP); and diastolic blood pressure (DBP; see S2 Appendix for descriptive statistics for the biomarkers). The *z* score method has previously performed well in a comparison of AL measurement methods [31]. For each biomarker, *z* scores were calculated using the standard formula, where the variable mean is subtracted from the observed values to centre the mean at zero, and then the values are divided by the variable standard deviation to standardise the variable by giving it a standard deviation of 1. To normalize their distributions, before biomarkers were *z* scored and summed, the following biomarkers were log transformed: CRP; fibrinogen; HbA1c; triglyceride; BMI; SBP; and DBP. For all biomarkers we used except HDL ratio, risk can be indicated with high or low values, so we used absolute *z* scores, which indexed how far values were from the sample mean. Risk is indicated with only a high HDL ratio, so *z* scores below zero on this biomarker were changed to zero before the biomarker *z* scores were combined to create the AL scores. AL scores were not computed for the 27 participants who had missing data on half or more of these biomarkers.

The following biomarkers were measured using blood samples: albumin; CRP; fibrinogen; HbA1c; HDLR; and triglycerides. Albumin was measured using Vitros ALB slides, with colourimetric tests conducted using the Vitros Fusion 5.1 FS and Vitros 4600 Chemistry Systems. CRP was measured with the OrthoFusion 5.1 F.S. analyser using dry immuno-rate slides (Vitros Chemistry Products CRP slides, Ortho Clinical Diagnostics, Buckinghamshire, UK). Fibrinogen was measured with an automated Clauss assay (TOPS coagulator, Instrumentation Laboratory, Warrington, UK). Non-fasting HbA1c was measured with an Adams HA-8160 HbA1c analyser, which uses a high performance liquid chromatography method. High-density lipoprotein and total cholesterol were measured using the Abbott Architect c16000 and the HDL ratio was calculated by dividing total cholesterol by high-density lipoprotein. Triglycerides were also measured using the Abbott Architect c16000.

BMI was computed by dividing participants’ weight in kilograms by their squared height in meters. SBP and DBP values were the mean of three sitting readings.

#### Medical history and medication status

During a medical interview at the time of the cognitive assessment and blood sampling, participants’ medical history was taken and they were asked to provide information on their current prescription medications. Adjustments were made to raw biomarker values where participants were taking drugs related to AL (antihypertensive, lipid-lowering, insulin and other diabetes medications) [62]. For those taking antihypertensives, SBP was increased by 10mmHG and DBP by 5mmHG. For those taking statins, total cholesterol was increased by 1.8mmol/l and CRP by 0.02mg/dl. Finally, for those taking insulin or other diabetes medication, HbA1c was increased by 1%.

#### Covariates

To control for their influence, sex, age at the day of assessment at each wave, and a count of medical conditions at each wave were used as covariates. Participants self-reported medical condition information during structured interviews. The following medical conditions were included in the count: arthritis; problems with blood circulation; history of cardiovascular disease; dementia; diabetes; high blood pressure; high cholesterol; leg pain when walking or in bed at night; cancer; Parkinson’s disease; history of stroke; thyroid disorder; and an additional point was added if a participant had another medical condition not specifically asked about. In response to a reviewer request, we also included years of education (collected at Wave 1) as a covariate and present results of this analysis in S4 Appendix.

### Statistical analysis

The R software environment (Version 3.2.0; [63]) was used for data importing, data management, data cleaning, plotting distributions, calculating descriptive statistics, calculating AL scores and facilitating model fitting. These tasks were aided by the R packages psych (Version 1.5.4; [64]), Hmisc (Version 3.16–0; [65]), memisc (Version 0.97; [66]), likert (Version 1.2; [67]), plyr (Version 1.8.3; [68]) and MplusAutomation (Version 0.6–3; [69]).

**Fig 1:**
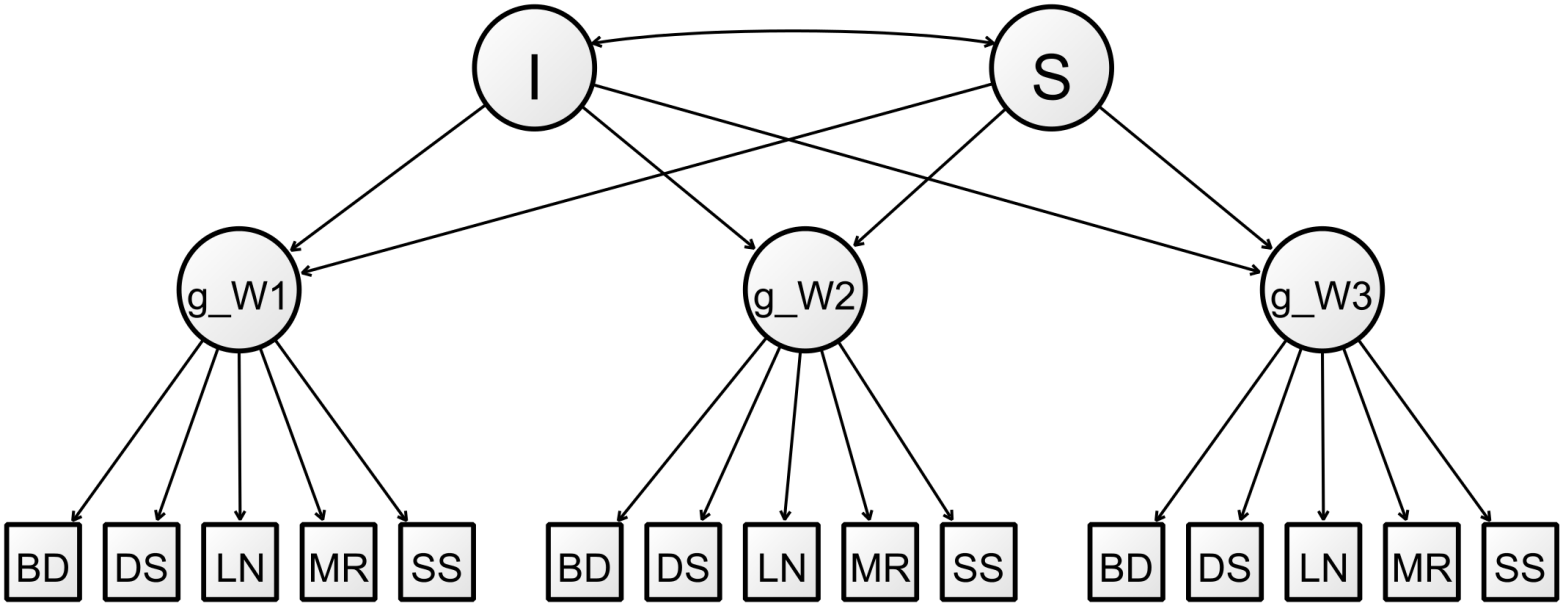
Path diagram of the unconditional LGCM for general cognitive ability in one group. Variances, residual covariances and estimates excluded for clarity. The timepoint of each item is indicated here by the factor the item loads on. I = intercept. S = slope. g_W1 = Wave 1 general cognitive ability. g_W2 = Wave 2 general cognitive ability. g_W3 = Wave 3 general cognitive ability. BD = Block Design. DS = Digit Symbol Coding. LN = Letter-Number Sequencing. MR = Matrix Reasoning. SS = Symbol Search.

Second-order multiple group latent growth curve models (MGLGCMs) using groups based on *APOE* genotype were fitted to answer the study’s research questions. Fig 1 is a diagram of the unconditional LGCM in one group. The observed cognitive test scores loaded on a latent factor representing cognitive ability at their respective waves, and the three measurements of the general cognitive ability factor loaded on the latent intercept and slope growth factors. The intercept growth factor represented initial level of ability and the slope growth factor represented linear change in ability over time. The mean of the intercept represents the average initial level of ability while the mean of the slope represents the average linear change in ability over time. The variances of the growth factors indicate how much variability there is around these means. We tested whether the intercept and slope means were related to neuroticism, depressive symptoms and allostatic load, and whether these relationships varied across genotypic groups.

The intercept growth factor’s loadings were fixed at 1.0. The slope growth factor’s loadings were fixed at values calculated to reflect the differences in mean ages between waves of assessment. The slope loadings were therefore fixed at 0.0, 1.0 and 2.27 on the general cognitive ability factors from Waves 1, 2 and 3 respectively.

#### Grouping variables

Primary analyses used groups of *APOE* E4 carriers and non-carriers. Following Dick et al. [70], all analyses were also conducted using groups of individuals possessing specific genotypes. The sample size of most genotypes was prohibitively small, so groups were formed of individuals with the two most common *APOE* genotypes, E3/E3 and E3/E4.

#### Measurement models and covariates

The measurement invariance of general cognitive ability across time and across groups and the measurement invariance of neuroticism across groups was then tested to establish the equivalence of the latent variables used for model estimation. After this, the main effect of *APOE* E4 was assessed by testing the invariance of the growth factor means across groups. For full details of these models, see S1 Appendix.

Next, covariates were added to the models. Sex was modeled as a time-invariant covariate predicting both growth factors. Age at each wave (coded in years and centered at 67) and number of medical conditions at each wave were modeled as time-varying covariates predicting all cognitive ability manifest variables at their respective waves. All covariances between covariates were freely estimated in each group, except for covariances between different time-varying covariates from different waves, which were fixed at zero. All estimated parameters involving covariates were freely estimated across groups. This effectively also controlled for gene-by-covariate interactions, in line with current methodological recommendations [71].

#### Effects of depressive symptoms, neuroticism and AL

Separate models were fitted in which depressive symptoms, neuroticism and AL predicted the cognitive ability intercept and slope. Additionally, a mediation model was fitted in which neuroticism predicted the growth factors both directly and through AL.

#### Model testing

The moderation of each effect of interest (i.e. the effects of depressive symptoms, neuroticism and AL on the initial level of cognitive ability [intercept] and the subsequent linear change in cognitive ability [slope]) was tested by comparing the fit of two models: one with the regression for the effect freely estimated in both the group of E4 non-carriers and the group of E4 carriers, one with it constrained to equality across groups. A significant decrement in fit in the latter model would indicate moderation.

**Fig 2:**
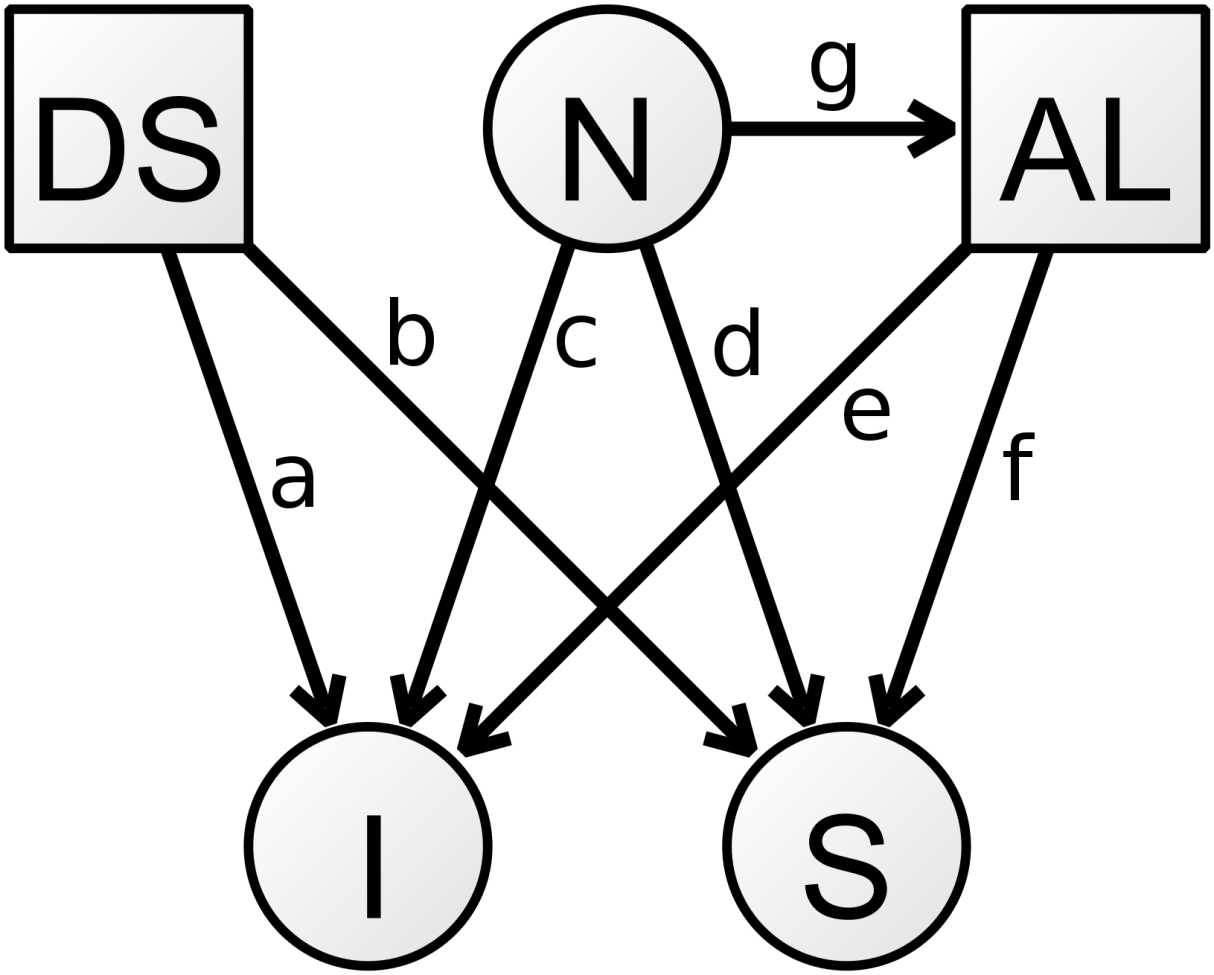
Structural model paths for the various hypothesis-testing models. Covariates, variable variances and the covariance between the intercept and slope omitted for clarity. Lower case letters are the path names referred to in text. DS = depressive symptoms. N = neuroticism. AL = allostatic load. I = cognitive ability intercept. S = cognitive ability slope.

Fig 2 illustrates the growth factors and predictors of interest for the models in one group. Note that the covariance between the intercept and slope has been omitted for clarity. In this figure, for example, paths a and b from depressive symptoms to the growth factors were only in the three models testing the (moderation of the) effects of depressive symptoms: one with both paths freely estimated across groups; one with the path to the intercept jointly estimated/constrained to equality across groups and the path to the slope freely estimated across groups; and one with the path to the slope jointly estimated/constrained to equality across groups and the path to the intercept freely estimated across groups. The (moderation of the) effects of neuroticism (paths c and d) and AL (paths e and f) were tested using the same setup, with the only paths included being c and d for the neuroticism models and e and f for AL models. Finally, the mediation models contained paths c-f. In the mediation model, it was the second stage of the model, from AL to cognitive ability and decline that was tested for moderation (paths e and f).

Covariances between predictors and covariates were freely estimated in each group, except those between predictors and age at the latter two waves, which were fixed at zero.

Full information maximum likelihood (FIML) estimation in Mplus Version 7.31 [72] was used to fit all SEMs. We assumed that missing data was missing at random, a condition under which FIML estimation performs well [73].

Mardia’s tests indicated that input variables were not multivariate normally distributed, so robust standard errors (*SE*s) were computed for all models using a sandwich estimator. As robust *SE*s were calculated, Satorra-Bentler scaled chi-square difference tests for nested models based on their loglikelihoods [74, 75] were used to compare models, with *p* ≤ .05 indicating a statistically significant difference in fit. The *SE*s for tests of the indirect effect in mediation models were calculated using the delta method.

#### Robustness checks

As a check on the robustness of our primary operationalization of neuroticism, models were also fitted using an alternative parceling scheme for neuroticism that had parcels comprised of items that were also included in facet scales for depression (items 19, 39, 49), anxiety (4, 9, 14, 24) and anger (29, 44) in an IPIP representation of the NEO Personality Inventory–Revised [76]. Plus, as a check on the influence that pathological cognitive aging may have had on results, additional models were fitted after excluding participants who scored below 24 on the MMSE (13 E4 non-carriers, 12 E4 carriers; 10 with the E3/E3 genotype, 7 with the E3/E4 genotype) and/or with those who reported having had a stroke or having dementia or Parkinson’s disease also excluded (92 E4 non-carriers, 62 E4 carriers; 82 with the E3/E3 genotype, 34 with the E3/E4 genotype). The 23v24 cutoff we used for the MMSE is common when screening for cognitive impairment [77].

#### Models controlling for education

Lastly, we fitted models that added years of education as a covariate predicting the cognitive ability intercept and slope, thus controlling for the effects of education (see S4 Appendix).

## Results

For descriptive statistics for the whole genotyped sample as well as for the groups of E4 non-carriers and E4 carriers, see Table 1. For raw biomarker descriptive statistics, descriptive statistics for the secondary grouping with groups of those with the E3/E3 and E3/E4 genotypes, and correlations within each group, see S2 Appendix. Results from the models using both groupings were similar, so here we present only the models from the more powerful analyses using the groups of E4 non-carriers and E4 carriers. For the results from models using groups of those with the E3/E3 and E3/E4 genotypes, see S3 Appendix. For the full measurement model results, see S1 Appendix.

**Table 1:** Descriptive Statistics for Groups of *APOE* E4 Non-carriers and Carriers.

Variable		*n*			*M*			*SD*			*Skew*			*Kurtosis*		
		both groups	no E4 group	E4 group	both groups	no E4 group	E4 group	both groups	no E4 group	E4 group	both groups	no E4 group	E4 group	both groups	no E4 group	E4 group
**Block Design**	**W1**	1023	719	304	33.73	33.99	33.13	10.30	10.24	10.42	0.27	0.28	0.25	-0.24	-0.17	-0.42
	**W2**	818	573	245	33.50	33.79	32.84	33.50	10.19	9.75	0.47	0.42	0.59	0.08	0.08	0.08
	**W3**	653	453	200	32.08	32.67	30.75	32.08	9.80	10.41	0.30	0.30	0.37	0.25	0.23	0.32
**Digit Symbol**	**W1**	1023	717	306	56.63	57.02	55.70	13.00	13.14	12.62	0.04	0.03	0.05	-0.24	-0.23	-0.29
	**W2**	816	572	244	56.34	56.65	55.60	12.41	12.66	11.82	0.05	0.07	-0.05	-0.19	-0.37	0.27
	**W3**	647	450	197	53.70	54.84	51.10	12.92	12.34	13.85	-0.10	-0.12	0.04	-0.09	0.02	-0.29
**Letter-Number Sequencing**	**W1**	1017	716	301	10.90	11.03	10.62	3.16	3.20	3.04	0.14	0.14	0.09	0.08	0.00	0.25
	**W2**	818	574	244	10.91	10.94	10.83	3.08	3.13	2.95	0.24	0.24	0.21	0.30	0.14	0.71
	**W3**	649	454	195	10.46	10.57	10.22	2.99	3.03	2.86	0.18	0.28	-0.11	0.35	0.49	-0.28
**Matrix Reasoning**	**W1**	1024	720	304	13.46	13.69	12.91	5.11	5.14	5.00	-0.02	-0.07	0.10	-1.00	-0.97	-1.05
	**W2**	818	573	245	13.12	13.25	12.83	4.99	5.06	4.82	-0.03	-0.07	0.07	-1.03	-1.00	-1.12
	**W3**	651	454	197	12.99	13.29	12.29	4.94	4.96	4.85	0.04	0.00	0.14	-0.95	-0.97	-0.90
**Symbol search**	**W1**	1024	719	305	24.71	25.06	23.90	6.40	6.19	6.80	-0.09	-0.03	-0.14	0.82	0.13	1.81
	**W2**	817	572	245	24.52	24.78	23.92	6.22	6.13	6.38	-0.31	-0.28	-0.36	0.70	0.64	0.75
	**W3**	649	453	196	24.55	25.45	22.49	6.48	6.15	6.77	-0.22	-0.23	-0.05	0.73	0.28	1.59
**Age**	**W1**	1028	722	306	69.53	69.53	69.54	0.83	0.82	0.85	-0.04	-0.05	0.00	-0.88	-0.86	-0.94
	**W2**	820	575	245	72.49	72.49	72.48	0.71	0.70	0.73	0.00	-0.03	0.06	-0.83	-0.77	-0.98
	**W3**	659	459	200	76.24	76.22	76.29	0.67	0.67	0.69	-0.05	-0.06	-0.02	-0.84	-0.70	-1.15
**Number of medical conditions**	**W1**	1020	719	301	1.64	2.89	2.88	1.64	1.66	1.58	0.49	0.54	0.34	0.19	0.24	-0.01
	**W2**	820	575	245	1.72	3.29	3.51	1.72	1.70	1.74	0.42	0.35	0.56	0.09	-0.33	0.87
	**W3**	652	453	199	1.76	3.89	3.94	1.76	1.78	1.70	0.48	0.33	0.88	0.42	-0.04	1.57
**Dep. symptoms**	**W1**	1023	718	305	2.79	2.84	2.68	2.22	2.20	2.28	1.35	1.20	1.66	2.36	1.40	4.40
**Neuroticism parcel**	**1**	912	639	273	2.13	2.14	2.11	0.83	0.81	0.88	0.59	0.50	0.76	-0.12	-0.25	0.07
	**2**	911	639	272	2.61	2.62	2.58	0.81	0.78	0.87	0.24	0.21	0.32	-0.15	-0.20	-0.16
	**3**	912	639	273	2.43	2.45	2.40	0.79	0.78	0.81	0.18	0.13	0.29	-0.37	-0.41	-0.30
**Allostatic load**	**W1**	1020	716	304	0.74	0.74	0.74	0.25	0.24	0.26	0.99	0.88	1.18	1.76	1.59	1.99
**Neuroticism facet parcels**	**Dep**	912	639	273	2.21	2.20	2.22	0.88	0.86	0.94	0.48	0.40	0.61	-0.32	-0.47	-0.17
	**Anx**	911	639	272	2.73	2.75	2.69	0.86	0.84	0.90	0.13	0.14	0.12	-0.38	-0.40	-0.39
	**Ang**	765	530	235	2.47	2.51	2.39	0.86	0.86	0.86	0.22	0.13	0.45	-0.76	-0.89	-0.36
		Females			Males											
		512	369	143	516	353	163									

W1 = Wave 1. W2 = Wave 2. W3 = Wave 3. Dep. symptoms = depressive symptoms. Dep = depression. Anx = anxiety. Ang = anger.

### Unconditional latent growth curve models

In initial models, the slope growth factor had a negative residual variance. The slope growth factor variance was therefore fixed at zero. Note that models with this restriction fitting well suggested that cognitive change in the sample was relatively homogeneous.

### Main effect of *APOE* genotype

Satorra-Bentler chi-square tests of the main effect of *APOE* genotype are in Table 2. Note that another study using this sample found that *APOE* E4 carriers had a lower level of initial cognitive ability and greater cognitive decline [49]. In the present study, the initial level of cognitive ability was 0.18 *SD* lower (*SE* = 0.078) in E4 carriers than non-carriers. There was not a statistically significant difference between groups in the amount of cognitive change. Sex, age and medical conditions were then added to the model as covariates.

**Table 2:** Tests of the Measurement Invariance of the Growth Factors in Groups of *APOE* E4 Non-carriers and Carriers.

Growth factor means constrained to be equal	χ^2^_SB_ (Δχ^2^_SB_)	*df* (Δχ^2^_SB_ *df*)	Δχ^2^_SB_ *p*	*RMSEA*	*SRMR*
**None**	517.58 (N/A)	192 (N/A)	N/A	.057	.068
**Intercept**	522.46 (4.84)	193 (1)	.028	.058	.070
**Slope**	521.14 (3.73)	193 (1)	.054	.058	.069

*N* = 1027. Non-E4 carriers group *n* = 721. E4 carriers group *n* = 306. All χ^2^_SB_ *p*s < .001. _SB_ = Satorra-Bentler. *RMSEA* = root mean square error of approximation. *SRMR* = standardized root mean square residual.

### Main effects of depressive symptoms, neuroticism and AL

Estimates of the main effects of depressive symptoms, neuroticism and AL on cognitive ability and decline, along with tests of the moderation of these effects, are in Table 3. As constraining the regressions of the cognitive growth factors on depressive symptoms, neuroticism and AL did not worsen fit, main effects were assessed primarily based on the regression coefficients jointly estimated across groups. Depressive symptoms (E4 non-carriers group [E4-] *β* [standardized coefficient] = -0.18, *p* < .001; E4 carriers group [E4+] *β* = -0.19, *p* < .001), neuroticism (E4- *β* = -0.24, *p* < .001; E4+ *β* = -0.27, *p* < .001) and AL (E4- *β* = -0.10, *p* = .005; E4+ *β* = -0.11, *p* = .006) all had small-to-moderate negative associations with the intercept. Depressive symptoms (E4- *β* = -0.88, *p* = .003; E4+ *β* = -1.00, *p* < .001) and AL (E4- *β* = -0.85, *p* = .018; E4+ *β* = -0.97, *p* = .019) were strongly related to the slope. Standard errors for the effect of neuroticism on the slope were large and this parameter was never statistically significant.

**Table 3:** Main Effect and Moderation Tests in Groups of *APOE* E4 Non-carriers and Carriers.

Predictor	Regression constrained	Regression of intercept on predictor *estimate* (*SE*)		Regression of slope on predictor *estimate* (*SE*)		χ^2^_SB_ (Δχ^2^_SB_)	*df* (Δχ^2^_SB_ *df*)	Δχ^2^_SB_ *p*	*RMSEA*	*SRMR*
		no E4 group	E4 group	no E4 group	E4 group					
**Depressive symptoms**	**None**	-0.17 (0.04)***	-0.20 (0.07)**	-0.91 (0.24)***	-0.98 (0.65)	789.96 (N/A)	380 (N/A)	N/A	.046	.069
**Depressive symptoms**	**Intercept**	-0.18 (0.04)***	-0.19 (0.04)***	-0.91 (0.24)***	-0.99 (0.47)*	789.88 (0.05)	381 (1)	.82	.046	.069
**Depressive symptoms**	**Slope**	-0.18 (0.04)***	-0.20 (0.07)**	-0.88 (0.30)**	-1.00 (0.06)***	790.48 (0.21)	381 (1)	.644	.046	.069
**Neuroticism**	**None**	-0.25 (0.05)***	-0.24 (0.07)***	-0.73 (0.67)	-0.57 (5.78)	919.86 (N/A)	472	N/A	.043	.065
**Neuroticism**	**Intercept**	-0.24 (0.04)***	-0.27 (0.04)***	-0.76 (0.59)	0.02 (7.23)	920.10 (0.29)	473 (1)	.59	.043	.065
**Neuroticism**	**Slope**	-0.25 (0.05)***	-0.23 (0.07)***	-0.65 (0.78)	-0.91 (1.17)	919.40 (0.00)	473 (1)	.83	.043	.065
**Allostatic load**	**None**	-0.10 (0.04)*	-0.09 (0.07)	-0.83 (0.42)	-0.98 (0.28)***	801.62 (N/A)	380 (N/A)	N/A	.046	.071
**Allostatic load**	**Intercept**	-0.10 (0.04)**	-0.11 (0.04)**	-0.83 (0.41)*	-0.98 (0.33)**	801.69 (0.08)	381 (1)	.77	.046	.071
**Allostatic load**	**Slope**	-0.10 (0.04)*	-0.09 (0.07)	-0.85 (0.36)*	-0.97 (0.41)*	801.40 (0.03)	381 (1)	.86	.046	.071

*N* = 1028. Non-E4 carriers group *n* = 722. E4 carriers group *n* = 306. All χ^2^_SB_ *p*s < .001. All estimates are standardized. _SB_ = Satorra-Bentler. *RMSEA* = root mean square error of approximation. *SRMR* = standardized root mean square residual.

*p ≤ .05.

**p ≤ .01.

***p ≤ .001.

### Moderation of the effects of depressive symptoms, neuroticism and AL

Satorra-Bentler scaled chi-square tests, which compared models where the effects of depressive symptoms, neuroticism and AL were and were not constrained to equality across groups, were all non-significant. This suggested that none of the associations of depressive symptoms, neuroticism and AL with the intercept and slope were moderated by *APOE* E4 status. The absence of moderation was also supported by the *RMSEA* and *SRMR* statistics, which suggested model fit was near-identical for models with and without regression constraints.

### Tests of mediation and moderated mediation

Lastly, models testing whether the association between neuroticism and cognitive ability and decline was mediated through AL were fitted. Parameter estimates from these models are in Table 4. Tests of mediation and moderation in the mediation models are in Table 5. All estimates of indirect effects were small and not statistically significant. Satorra-Bentler scaled chi-square tests were non-significant, which suggested that the paths from AL to the cognitive intercept and slope were not moderated by *APOE* E4 status. A lack of moderation was also indicated by the *RMSEA* and *SRMR* model statistics, which were identical to three decimal places in mediation models with and without regression constraints.

**Table 4:** Parameter Estimates from the Mediation Model in Groups of APOE E4 Non-carriers and Carriers.

Regression constrained to equality	Intercept on N *est*. (*SE*)		Slope on N *est*. (*SE*)		AL on N *est*. (*SE*)		Intercept on AL *est*. (*SE*)		Slope on AL *est*. (*SE*)	
	no E4 group	E4 group	no E4 group	E4 group	no E4 group	E4 group	no E4 group	E4 group	no E4 group	E4 group
**None**	-0.24 (0.05)***	-0.23 (0.07)**	-0.44 (0.59)	-0.10 (1.43)	0.08 (0.04)	0.08 (0.07)	-0.09 (0.04)*	-0.07 (0.06)	-0.72 (0.47)	-0.98 (0.23)***
**Intercept on AL**	-0.24 (0.05)***	-0.23 (0.07)**	-0.43 (0.58)	-0.11 (1.55)	0.08 (0.04)	0.08 (0.07)	-0.09 (0.04)*	-0.09 (0.04)*	-0.73 (0.45)	-0.98 (0.28)***
**Slope on AL**	-0.24 (0.05)***	-0.23 (0.07)**	-0.41 (0.57)	-0.14 (1.86)	0.08 (0.04)	0.08 (0.07)	-0.09 (0.04)*	-0.07 (0.07)	-0.75 (0.41)	-0.96 (0.38)*

*N* = 1028. Non-E4 carriers group *n* = 722. E4 carriers group *n* = 306. All estimates are standardized. N = neuroticism. AL = allostatic load.

*p ≤ .05.

**p ≤ .01.

***p ≤ .001.

**Table 5:** Tests of Mediation and Moderated Mediation in Groups of APOE E4 Non-carriers and Carriers.

Regression constrained to equality	Indirect effect on intercept *est*. (*SE*)		Indirect effect on slope *est*. (*SE*)		χ^2^_SB_ (Δχ^2^_SB_)	*df* (Δχ^2^_SB_ *df*)	Δχ^2^_SB_ *p*	*RMSEA*	*SRMR*
	no E4 group	E4 group	no E4 group	E4 group					
**None**	-0.007 (0.005)	-0.006 (0.007)	-0.056 (0.049)	-0.078 (0.070)	962.84 (N/A)	508 (N/A)	N/A	.042	.063
**Intercept on AL**	-0.006 (0.004)	-0.007 (0.007)	-0.058 (0.048)	-0.076 (0.070)	962.92 (0.10)	509 (1)	.75	.042	.063
**Slope on AL**	-0.007 (0.005)	-0.006 (0.007)	-0.059 (0.046)	-0.076 (0.071)	962.44 (0.04)	509 (1)	.85	.042	.063

*N* = 1028. Non-E4 carriers group *n* = 722. E4 carriers group *n* = 306. All *n*s for E4 carriers group = 306. All χ^2^_SB_ *p*s < .001. All estimates are standardized. _SB_ = Satorra-Bentler. *RMSEA* = root mean square error of approximation. *SRMR* = standardized root mean square residual.

#### Main effects in mediation models

In the mediation models, neuroticism had a statistically significant small-to-moderate negative association with the intercept. Standard errors for estimates of the effect of neuroticism on the slope were large and this parameter was not significant in any mediation models. Neuroticism did not significantly predict AL in either group in any model. When the regression of the intercept on AL was jointly estimated, AL had a small significant negative association with the intercept in both groups. When the regression of the slope on AL was jointly estimated, AL significantly predicted the slope in the E4 carriers group.

### Robustness checks

The only notable difference between the main model and robustness check model results was that constraining the intercept growth factor means to be equal in the *APOE* E4 status groups did not cause a statistically significant decrement in fit when the aforementioned participants were excluded: Δχ^2^_SB_(1, *N* = 1002) = 3.39, *p* = .066. In concordance with the main model results, Satorra-Bentler scaled chi-square tests indicated that the effects of neuroticism, depressive symptoms and AL on cognitive ability and decline were not moderated by *APOE* genotype in these models. Parameter estimates and their statistical significance did not systematically differ between the main and robustness check models.

### Models controlling for education

Full tables of results can be found in S4 Appendix (Tables N-P). The average years of education were similar across the groups of *APOE* E4 non-carriers (*M* [*SD*] = 10.74 [1.10], *Mdn* = 10, *range* = 8–13, *skew* = 0.79, *kurtosis* = -0.41) and carriers (*M* [*SD*] = 10.74 [1.19], *Mdn* = 10, *range* = 7–14, *skew* = 0.71, *kurtosis* = -0.16).

Overall, the results from these models were similar to the results from models without education as a covariate. Most notably, the effects of depressive symptoms, neuroticism and AL on baseline cognitive ability tended to be attenuated by around 20–30% (maximum Δ = 0.07). Consequently, the effect of AL on baseline cognitive ability only remained statistically significant in the model in which this regression was jointly estimated across groups. Also, in the non-E4 carriers group, there were large attenuations for the regressions of cognitive change on depressive symptoms (maximum Δ = 0.39) and AL (maximum Δ = 0.41), while in the E4 carriers group, the standard errors tended to be much larger for all effects on cognitive change. This meant that with one exception, these effects were no longer statistically significant. In the mediation models, estimates for the regression of AL on neuroticism were very similar, but this path did become statistically significant in the E4 non-carriers group in each model.

Crucially, like in models without education as a covariate, *APOE* E4 status did not moderate the effects of depressive symptoms, neuroticism and AL on baseline cognitive ability and subsequent cognitive decline. Plus, importantly, AL did not mediate the effects of neuroticism on cognitive ability and decline.

## Discussion

The current study sought to test five research questions on the relationships depressive symptoms, neuroticism and AL have with baseline cognitive ability and subsequent cognitive decline. The first two of these questions replicated previously reported main effects. At baseline, *APOE* E4 carriers had significantly lower cognitive ability, but the rate of cognitive decline did not significantly differ between E4 non-carriers and E4 carriers. When fitting these models, the variance of the slope growth factor had to be constrained to zero, which indicated that there was very little variability in cognitive change across the sample.

Our study also replicated the relationship between higher AL and greater subsequent cognitive decline. This replication was important because all previous studies on this relationship used data from the MacArthur Successful Aging Study [46–48]. We also replicated previous findings that depressive symptoms are related to cognitive decline. Plus, as in previous studies using this sample, AL [45] and depressive symptoms [52] were linked to contemporaneously measured cognitive ability. However, we failed to replicate prior findings that the associations between depressive symptoms and neuroticism and cognitive ability and decline are moderated by *APOE* E4.

In the current study, we conducted moderated-mediation analyses investigating the hypothesis that AL mediates associations between neuroticism and cognitive outcomes. Results from a novel mediation model suggested that the associations of neuroticism with cognitive ability and decline were not mediated through AL. Further, paths in the second stage of this mediation model, from AL to cognitive ability and decline, were not moderated by *APOE* E4. Paths from AL to cognitive ability and decline were also not moderated by *APOE* E4 in models without neuroticism.

Given the varied findings in this and other studies, it is important to consider the features that differentiate the studies as these may explain the differences in results. With respect to the moderating effect of *APOE* E4 status, an important between-study variable is the measurement of key phenotypes. Studies that have used multiple cognitive tests and non-dichotomous depressive symptomatology measures have tended not to find the interaction (the present study, [22, 23]; cf. [21]), whereas those that have used a cognitive impairment screener or clinical diagnosis of cognitive impairment along with a dichotimization or clinical diagnosis of depressive symptomatology have tended to find the interaction [12–18]. Our study also contrasts with most of those that have found the interaction in that depression, MCI and dementia were not prevalent in our sample, and the cognitive decline observed was not substantial. It may be, then, that *APOE* E4 moderates the influence of depressive symptomatology on pathological but not non-pathological cognitive aging. This would have implications for future research in this area and how research would inform clinical practice.

With regard to the interaction of neuroticism and *APOE* E4, Dar-Nimrod et al. [25, 26] measured cognitive ability using a cognitive impairment test and found that *APOE* moderated the influence of neuroticism on cognitive ability. This also contrasts with the cognitive ability measurement and findings of the present study. Results from other studies on the *APOE*-by-neuroticism interaction suggest that the specific cognitive domains studied should also be considered. Sapkota et al. found the *APOE*-by-neuroticism interaction did not predict baseline or subsequent change in declarative memory [24]. Chapman et al. found an overall *APOE*-by-neuroticism interaction in a multivariate analysis of variance with five cognitive domains as outcomes [27]. However, the interaction was only statistically significant for one specific domain: attention [27].

In the current study, we operationalized cognitive ability using multiple tests and a latent variable modeling perspective. However, these tests were largely executive functioning tasks, and thus our test battery did not contain many tests tapping, for example, memory performance. To the extent that cognitive screening tools cover more aspects of cognition in a more general way, it may be that results differing results suggest pathways of impact via specific abilities.

Consistent with the findings of the main models, in models that controlled for the effect of education on baseline cognitive ability and decline, *APOE* E4 did not moderate any effects and AL did not mediate the relationships neuroticism had with cognitive ability and decline. Main effects on baseline cognitive ability and cognitive decline were attenuated in these models, which suggested that education’s prediction of cognitive outcomes in older age may overlap with that of neuroticism, depressive symptoms and AL. We chose not to control for education in our main models because it is strongly related to early life cognitive ability, which itself is strongly related to older age cognitive ability [78]. Controlling for education, therefore, changed the interpretation of the focal parameters, meaning these models answered research questions that were somewhat different to those we set out to answer.

The ideal design for a future longitudinal study into this interaction would include comprehensive and multifaceted measures of depressive symptomatology and cognitive ability, along with clinical diagnoses of depression and cognitive disorders. This would enable the interaction to be simultaneously assessed in relation to both healthy and pathological cognitive aging. For adequate statistical power, a larger sample of MCI and dementia cases would be required than were present in the current study’s sample. In addition, researchers could collect further data to investigate potential mechanisms, such as mediators in the brain. It is possible that the interaction functions through depressive symptoms leading to increased amyloid-beta plaques [79] and/or decreased hippocampal volume [80], and these effects in the brain are then have stronger negative associations with worse cognitive ability in E4 allele carriers [81, 82].

This study also found that the association between neuroticism and cognitive ability was not mediated through AL as has previously been proposed in the literature. In these mediation models, neuroticism was not linked to AL, except in the additional models where education was added as a covariate. The present study is one of the first to relate AL and a personality domain. A previous study found that in models with demographic covariates, AL had a small positive effect on baseline neuroticism but did not predict subsequent four-year change in neuroticism [40]. The relationships between AL and personality factors may prove an interesting area for future research. All mediation models suggested that, in this sample, AL does not mediate the relationships neuroticism has with cognitive ability and decline.

The index of AL used here consisted of biomarkers related to inflammation and metabolism, so "inflammatory and metabolic processes" ([25], p. 1152] seem to be an unlikely mechanistic mediator explaining Dar-Nimrod et al.’s [25, 26] *APOE*-by-neuroticism interaction. However, the present study was unable to test a potential pathway through HPA axis dysregulation given the biomarkers used to construct our measure of AL. Thus, such a pathway remains a possibility. A future investigation could test this study’s mediation model with cortisol replacing AL, because this particular biomarker has been related to neuroticism [36] and its associations with cognitive ability [41] and decline [42] have been found to be moderated by *APOE* E4 status.

In general, it is important to consider what processes and systems are reflected by measures of AL, and to what extent the variance in them is preserved. As noted, here the measure of AL did not contain any HPA axis markers, and so results must be considered in light of this. A strength of the AL operationalization used was that the *z* score sum method better preserves variance than the more commonly used sample quantile-based operationalizations [31]. A crucial step in integrating the growing body of research utilising the AL framework will be a comprehensive study of the impact of different biomarker sets and scoring methods.

A strength of this study is that groups based on both *APOE* E4 status and specific *APOE* genotypes were used. Plus, using SEM enabled error-free measurement of neuroticism and general cognitive ability. However, the availability of only three waves of data precluded the modeling of non-linear change and limited the statistical power to detect influences on and group differences in the slope growth factor. Here, cognitive ability was the only construct modeled longitudinally. Future research could also model depressive symptoms, neuroticism and AL data longitudinally. This would enable testing of the moderation of relationships between change in cognitive ability and change in depressive symptoms, neuroticism and AL. Another limitation of the present study is that no item- or subscale-level data were available for depressive symptoms. This meant that error-free measurement of depressive symptoms and tests focusing on facets of depression, like those performed by Rajan et al. [21], were not possible. Where possible, future studies on this topic should also investigate the moderation of relationships at the facet level of depression and neuroticism.

## Supporting information

S1 Appendix Measurement model details. Methods and results for the measurement models and unconditional latent growth curve models in both groupings.
(DOCX)

S2 Appendix Descriptive statistics and correlation matrices. Descriptive statistics for the groups of those with the *APOE* E3/E3 and E3/E4 genotypes, and correlations within all four groups.
(DOCX)

S3 Appendix Results in groups of those with the *APOE* E3/E3 and E3/E4 genotypes. Main and moderation effect test results, including for mediation models, in groups of those with the *APOE* E3/E3 and E3/E4 genotypes.
(DOCX)

S4 Appendix Results from models with education added as a covariate. Main and moderation effect test results, including for mediation models, in models where years of education was added as a predictor of the general cognitive ability intercept and slope.
(DOCX)
