# Supplementary material for: Apolipoprotein E genotype does not moderate the associations of depressive symptoms, neuroticism and allostatic load with cognitive ability and cognitive aging in the Lothian Birth Cohort 1936
Source: PLoS One. 2018 Feb 16;13(2):e0192604. doi: 10.1371/journal.pone.0192604 (PMC5815580; doi:10.1371/journal.pone.0192604)
Supplement: S1 Appendix — Methods and results for the measurement models and unconditional latent growth curve models in both groupings. (DOCX) [file pone.0192604.s001.docx]

**Measurement model details**

**Methods**

A path diagram of the unconditional LGCM for one group is shown in Figure A. The model pictured is unconditional because the growth factors are not conditioned on any predictors. The current study used second-order LGCMs. This means that manifest variables loaded on a latent factor representing general cognitive ability at their respective waves, and the three measurements of the general cognitive ability factor loaded on the latent intercept and slope factors. The intercept growth factor represents participants' initial level of general cognitive ability. It was fixed to load at 1.0 on the general cognitive ability factors from all waves of data. The slope growth factor represents linear change in general cognitive ability over time.

**
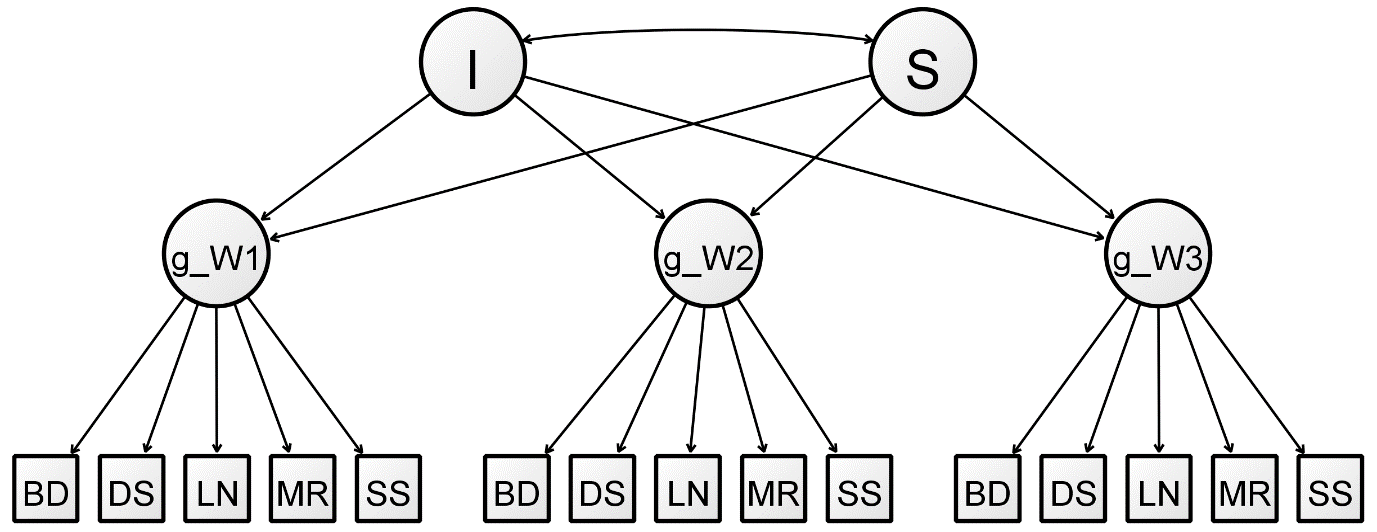
**

**Fig A.** Path diagram of the unconditional LGCM for general cognitive ability in one group. Variances, residual covariances and estimates excluded for clarity. The timepoint of each item is indicated here by the factor the item loads on. I = intercept. S = slope. g_W1 = Wave 1 general cognitive ability. g_W2 = Wave 2 general cognitive ability. g_W3 = Wave 3 general cognitive ability. BD = Block Design. DS = Digit Symbol Coding. LN = Letter-Number Sequencing. MR = Matrix Reasoning. SS = Symbol Search.

Taking into consideration model complexity, sample size and previous research [e.g,.^[[1]](#endnote-1)^], different acceptable fit cutoffs were chosen for the unconditional LGCMs for cognitive ability (root mean square error of approximation (*RMSEA*) ≤ .08; standardized root mean square residual (*SRMR*) ≤ .10) and the measurement model for neuroticism (*RMSEA* ≤ .05; *SRMR* ≤ .08; comparative fit index (*CFI*) ≥ .97). When testing measurement invariance, we compared models using Satorra-Bentler scaled chi-square tests. We also took into consideration the Akaike Information Criterion and Bayesian Information Criterion, for which a lower value indicates a model is preferred to, and differences in *RMSEA* and *SRMR*, for which the following cutoffs were taken as indicating noninvariance [^[[2]](#endnote-2)^]: Δ*RMSEA* ≥ .010; Δ*SRMR* ≥ .005.

**Statistical analysis**

Measurement models were fitted to establish measurement invariance of general cognitive ability and neuroticism over time and across groups. For cognitive ability, unconditional LGCMs with invariance of factor loadings and item intercepts over time were fitted in each group. For the first-order general cognitive ability factor at each wave, the first loading at each time point was fixed at 1.0 to identify the model, and covariances were estimated between the residuals of repeated measures of the same tests. Adjustments were made until acceptable, well-fitting models were achieved. Next, an unconditional MGLGCM was fitted. It included longitudinal measurement invariance within groups but no measurement constraints across groups. Then, metric invariance was tested by constraining factor loadings to be equal across groups and assessing the resulting change in model fit. Lastly, scalar invariance was tested by constraining manifest variable intercepts to equality across groups and observing the change in fit.

The main effects of *APOE* on cognitive ability and cognitive decline were then tested by comparing the fit of a model with unconstrained growth factor means to models with equality constraints on the intercept and slope means respectively. In subsequent models, so that the main effect of *APOE* genotype was included, the growth factor means were not constrained to be equal across groups.

For neuroticism, the initial measurement models fitted enforced metric and scalar invariance across groups. For identification purposes, the first loading in each model was fixed at 1.0. Primary analyses used three item parcels, each of which contained items about different facets of neuroticism. The parcels included three (4, 34, 49), four (9, 14, 29, 39) and three (19, 24, 44) items respectively [^[[3]](#endnote-3)^]. An individual's score on each parcel was the mean score of the items they answered, or coded as missing if they had not answered half or more of the parcel's items.

**Results**

**Cognitive ability measurement models**

First, unconditional LGCMs were fitted separately in each group. In initial models, there were negative variances for the slope in the non-E4 groups and the Wave 2 general cognitive ability factor in the E4 groups. These two variances were not statistically significant in any group. Therefore, these two variances were fixed at zero in both groups in all subsequent models. This produced models with acceptable fit.

MGLGCMs with invariance of indicator loadings and intercepts across time were then fitted. Results from the tests of measurement invariance of general cognitive ability are in Tables A and B. A marginally significant (*p* = .0485) Satorra-Bentler scaled chi-square test suggested that intercepts may not be invariant in the E4 status grouping. However, various fit index results were also considered and overall, the results suggested that intercepts were sufficiently invariant. Invariance of indicator loading and intercepts across groups was therefore retained in subsequent models. Unstandardized estimates from the final models are in Table C.

| **Table A. Results from Tests of Metric and Scalar Measurement Invariance of General Cognitive Ability Across Groups of *APOE* E4 Non-carriers and Carriers.** | | | | | | | |
| --- | --- | --- | --- | --- | --- | --- | --- |
| **Model** | **χ^2^_SB_ (Δχ^2^_SB_)** | ***df***  **(Δχ^2^_SB_ *df*)** | **Δχ^2^_SB_ *p*** | ***RMSEA*** | ***SRMR*** | ***AIC*** | ***BIC*** |
| **No metric or scalar MI across groups** | 505.44 (N/A) | 184  (N/A) | N/A | .058 | .064 | 74550.25 | 74974.61 |
| **Metric MI across groups** | 511.23 (6.10) | 188  (4) | .19 | .058 | .067 | 74548.94 | 74953.56 |
| **Scalar MI across groups** | 522.46 (11.14) | 193  (5) | .049 | .058 | .070 | 74550.00 | 74929.95 |
| *N* = 1027. Non-E4 carriers group *n* = 721. E4 carriers group *n* = 306. All χ^2^_SB_ *p*s < .001. _SB_ = Satorra-Bentler. *RMSEA* = root mean square error of approximation. *SRMR* = standardized root mean square residual. *AIC* = Akaike Information Criterion. *BIC* = Bayesian Information Criterion. | | | | | | | |

| **Table B. Results from Tests of Metric and Scalar Measurement Invariance of General Cognitive Ability Across Groups of Those with the *APOE* E3/E3 and E3/E4 Genotypes.** | | | | | | | |
| --- | --- | --- | --- | --- | --- | --- | --- |
| **Model** | **χ^2^_SB_ (Δχ^2^_SB_)** | ***df***  **(Δχ^2^_SB_ *df*)** | **Δχ^2^_SB_ *p*** | ***RMSEA*** | ***SRMR*** | ***AIC*** | ***BIC*** |
| **No metric or scalar MI across groups** | 500.19 (N/A) | 184  (N/A) | N/A | .063 | .069 | 62228.63 | 62637.53 |
| **Metric MI across groups** | 504.50 (5.28) | 188  (4) | .26 | .063 | .074 | 62226.92 | 62616.80 |
| **Scalar MI across groups** | 512.09 (7.51) | 193  (5) | .19 | .062 | .077 | 62224.42 | 62590.52 |
| *N* = 858. E3/E3 group *n* = 596. E3/E4 group *n* = 262. All χ^2^_SB_ *p*s < .001. _SB_ = Satorra-Bentler. *RMSEA* = root mean square error of approximation. *SRMR* = standardized root mean square residual. *AIC* = Akaike Information Criterion. *BIC* = Bayesian Information Criterion. | | | | | | | |

| **Table C. Unstandardized Estimates from Unconditional Multiple Group Latent Growth Curve Models for General Cognitive Ability.** | | | | |
| --- | --- | --- | --- | --- |
|  | ***Estimate* (*SE*)** | | | |
|  | **no E4 group** | **E4 group** | **E3/E3 group** | **E3/E4 group** |
| **Item loadings** |  |  |  |  |
| **BD W1/W2/W3** | 1.00 (0.00) | 1.00 (0.00) | 1.00 (0.00) | 1.00 (0.00) |
| **DS W1/W2/W3** | 1.46 (0.08) | 1.46 (0.08) | 1.45 (0.08) | 1.45 (0.08) |
| **LN W1/W2/W3** | 0.26 (0.01) | 0.26 (0.01) | 0.26 (0.02) | 0.26 (0.02) |
| **MR W1/W2/W3** | 0.44 (0.02) | 0.44 (0.02) | 0.44 (0.02) | 0.44 (0.02) |
| **SS W1/W2/W3** | 0.72 (0.04) | 0.72 (0.04) | 0.71 (0.04) | 0.71 (0.04) |
| **Item intercepts** |  |  |  |  |
| **BD W1/W2/W3** | 34.14 (0.35) | 34.14 (0.35) | 34.34 (0.38) | 34.34 (0.38) |
| **DS W1/W2/W3** | 57.11 (0.46) | 57.11 (0.46) | 57.07 (0.50) | 57.07 (0.50) |
| **LN W1/W2/W3** | 11.00 (0.10) | 11.00 (0.10) | 11.00 (0.11) | 11.00 (0.11) |
| **MR W1/W2/W3** | 13.64 (0.16) | 13.64 (0.16) | 13.61 (0.18) | 13.61 (0.18) |
| **SS W1/W2/W3** | 25.33 (0.21) | 25.33 (0.21) | 25.35 (0.23) | 25.35 (0.23) |
| **Item variances** |  |  |  |  |
| **BD W1** | 60.86 (3.80) | 59.34 (5.43) | 60.79 (4.33) | 61.51 (6.26) |
| **DS W1** | 74.55 (5.08) | 86.35 (9.05) | 74.50 (5.72) | 87.56 (9.78) |
| **LN W1** | 6.85 (0.41) | 5.98 (0.57) | 6.65 (0.45) | 6.02 (0.61) |
| **MR W1** | 16.52 (0.90) | 15.95 (1.22) | 17.37 (1.03) | 16.18 (1.33) |
| **SS W1** | 14.71 (1.24) | 20.38 (3.15) | 15.22 (1.41) | 21.30 (3.31) |
| **BD W2** | 62.31 (4.26) | 54.00 (5.66) | 64.96 (4.96) | 48.61 (5.75) |
| **DS W2** | 66.57 (4.92) | 71.38 (8.17) | 66.24 (5.53) | 73.79 (9.14) |
| **LN W2** | 6.63 (0.44) | 6.39 (0.64) | 6.15 (0.44) | 5.51 (0.59) |
| **MR W2** | 17.45 (0.97) | 14.27 (1.16) | 17.49 (1.08) | 15.14 (1.31) |
| **SS W2** | 15.51 (1.50) | 17.63 (2.15) | 16.22 (1.72) | 18.45 (2.45) |
| **BD W3** | 53.67 (4.61) | 47.72 (6.47) | 54.37 (5.20) | 46.94 (7.11) |
| **DS W3** | 62.45 (5.62) | 98.99 (11.96) | 60.44 (6.17) | 107.01 (13.74) |
| **LN W3** | 6.71 (0.52) | 5.58 (0.56) | 5.84 (0.48) | 5.77 (0.63) |
| **MR W3** | 15.98 (0.99) | 14.07 (1.53) | 15.93 (1.10) | 13.46 (1.57) |
| **SS W3** | 15.15 (1.56) | 19.09 (2.80) | 15.56 (1.78) | 19.60 (3.17) |
| **Item covariances** |  |  |  |  |
| **BD W1-BD W2** | 36.53 (3.30) | 37.11 (4.80) | 36.92 (3.76) | 36.63 (5.21) |
| **BD W1-BD W3** | 36.46 (3.29) | 30.12 (5.32) | 37.27 (3.72) | 29.14 (5.97) |
| **BD W2-BD W3** | 34.68 (3.32) | 30.06 (5.39) | 36.15 (3.79) | 30.01 (5.74) |
| **DS W1-DS W2** | 48.16 (4.50) | 57.68 (7.76) | 47.87 (5.18) | 59.40 (8.41) |
| **DS W1-DS W3** | 42.74 (4.60) | 53.92 (8.61) | 43.06 (5.24) | 55.99 (9.59) |
| **DS W2-DS W3** | 43.58 (4.67) | 55.32 (8.45) | 41.90 (5.33) | 61.74 (9.39) |
| **LN W1-LN W2** | 3.17 (0.33) | 2.77 (0.47) | 2.80 (0.34) | 2.76 (0.49) |
| **LN W1-LN W3** | 2.63 (0.35) | 1.99 (0.42) | 2.21 (0.35) | 1.99 (0.46) |
| **LN W2-LN W3** | 3.68 (0.40) | 3.31 (0.43) | 2.99 (0.34) | 3.08 (0.46) |
| **MR W1-MR W2** | 8.46 (0.82) | 5.92 (1.10) | 8.89 (0.94) | 6.01 (1.25) |
| **MR W1-MR W3** | 7.11 (0.89) | 7.86 (1.30) | 7.22 (1.01) | 7.13 (1.35) |
| **MR W2-MR W3** | 8.25 (0.91) | 5.74 (1.16) | 8.56 (1.01) | 5.92 (1.30) |
| **SS W1-SS W2** | 3.74 (1.08) | 5.23 (1.63) | 4.18 (1.28) | 6.77 (1.67) |
| **SS W1-SS W3** | 2.61 (1.05) | 2.82 (2.02) | 3.16 (1.24) | 3.89 (2.31) |
| **SS W2-SS W3** | 3.57 (1.15) | 5.48 (1.73) | 3.47 (1.31) | 6.37 (1.97) |
| **Factor means** |  |  |  |  |
| **Intercept** | 0.00 (0.00) | -1.15 (0.51) | 0.00 (0.00) | -0.82 (0.55) |
| **Slope** | -1.05 (0.08) | -1.35 (0.15) | -1.07 (0.09) | -1.35 (0.18) |
| **Factor variances** |  |  |  |  |
| **Intercept** | 44.75 (4.12) | 43.22 (5.47) | 45.99 (4.83) | 39.27 (5.71) |
| **Slope** | 0.00 (0.00) | 0.00 (0.00) | 0.00 (0.00) | 0.00 (0.00) |
| ***g* W1** | 1.62 (0.72) | 3.71 (1.60) | 1.35 (0.80) | 2.97 (1.76) |
| ***g* W2** | 0.00 (0.00) | 0.00 (0.00) | 0.00 (0.00) | 0.00 (0.00) |
| ***g* W3** | 2.02 (0.71) | 8.80 (3.11) | 2.23 (0.83) | 9.97 (3.63) |
| BD = Block Design. DS = Digit Symbol Coding. LN = Letter-Number Sequencing. MR = Matrix Reasoning. SS = Symbol Search. *g* = general cognitive ability. W1 = Wave 1. W2 = Wave 2. W3 = Wave 3. | | | | |

**Neuroticism measurement models**

Multiple group measurement models for neuroticism, using both parceling schemes, fit well in both groupings. Unstandardized estimates from the models using the primary parceling scheme are in Table D. Squaring standardized factor loadings showed that most of the variance, between 74% and 78%, was explained in the median item in each group. For the model in the E4 status groups, model fit was as follows: χ^2^(4, *N* = 913) = 1.34, *p* = .855, *RMSEA* = .000, *SRMR* = .015, *CFI* = 1.00. For the same model in the E3/E3 and E3/E4 groups: χ^2^(4, *N* = 759) = 1.49, *p* = .828, *RMSEA* = .000, *SRMR* = .016, *CFI* = 1.00.

| **Table D. Unstandardized Estimates from Multiple Group Neuroticism Measurement Models.** | | | | |
| --- | --- | --- | --- | --- |
|  | ***Estimate* (*SE*)** | | | |
|  | **no E4 group** | **E4 group** | **E3/E3 group** | **E3/E4 group** |
| **Item loadings** |  |  |  |  |
| **Parcel 1** | 1.00 (0.00) | 1.00 (0.00) | 1.00 (0.00) | 1.00 (0.00) |
| **Parcel 2** | 0.99 (0.04) | 0.99 (0.04) | 0.99 (0.04) | 0.99 (0.04) |
| **Parcel 3** | 0.80 (0.03) | 0.80 (0.03) | 0.82 (0.04) | 0.82 (0.04) |
| **Item intercepts** |  |  |  |  |
| **Parcel 1** | 2.15 (0.03) | 2.15 (0.03) | 2.14 (0.03) | 2.14 (0.03) |
| **Parcel 2** | 2.62 (0.03) | 2.62 (0.03) | 2.62 (0.03) | 2.62 (0.03) |
| **Parcel 3** | 2.44 (0.03) | 2.44 (0.03) | 2.43 (0.03) | 2.43 (0.03) |
| **Item variances** |  |  |  |  |
| **Parcel 1** | 0.17 (0.02) | 0.18 (0.03) | 0.16 (0.02) | 0.19 (0.03) |
| **Parcel 2** | 0.15 (0.02) | 0.15 (0.03) | 0.16 (0.02) | 0.15 (0.03) |
| **Parcel 3** | 0.31 (0.02) | 0.26 (0.03) | 0.27 (0.02) | 0.24 (0.03) |
| **Factor means** |  |  |  |  |
| **Neuroticism** | 0.00 (0.00) | -0.04 (0.06) | 0.00 (0.00) | -0.01 (0.06) |
| **Factor variances** |  |  |  |  |
| **Neuroticism** | 0.48 (0.04) | 0.62 (0.06) | 0.49 (0.04) | 0.61 (0.07) |

**Unconditional latent growth curve models**

Models with a slope variance of zero fitting well suggested that age-related cognitive decline was relatively homogeneous. Note that fixing the Wave 2 general cognitive ability (residual) variance at zero meant that all of the variance in this factor was explained by the growth factors. At least 92% of the variance in the general cognitive ability factors was explained by the growth factors, except for the Wave 3 factor in the E4 carriers and E3/E4 groups, in which 82.9% and 79.8% of the variance was explained respectively. Squaring standardized factor loadings showed that between 31.8% and 62.6% of the variance in manifest variables was explained by the general cognitive ability factors.

Table E contains the results from the tests of the invariance cognitive ability intercept and slope means across the groups of those with the E3/E3 and E3/E4 genotypes. There was no statistically significant decrement in fit when constraining the average initial level of ability or the average level of cognitive change to be jointly estimated across groups. This means there was no main effect of E4 allele possession on cognitive ability in this grouping.

| **Table E. Tests of the Measurement Invariance of the Growth Factors in Groups of Those with the *APOE* E3/E3 and E3/E4 Genotypes.** | | | | | |
| --- | --- | --- | --- | --- | --- |
| **Growth factor means constrained to be equal** | **χ^2^_SB_ (Δχ^2^_SB_)** | ***df* (Δχ^2^_SB_ *df*)** | **Δχ^2^_SB_ *p*** | ***RMSEA*** | ***SRMR*** |
| **None** | 509.97 (N/A) | 192 (N/A) | N/A | .062 | .076 |
| **Intercept** | 512.09 (2.13) | 193 (1) | .14 | .062 | .077 |
| **Slope** | 512.46 (2.47) | 193 (1) | .12 | .062 | .076 |
| *N* = 858. E3/E3 group *n* = 596. E3/E4 group *n* = 262. All χ^2^_SB_ *p*s < .001. _SB_ = Satorra-Bentler. *RMSEA* = root mean square error of approximation. *SRMR* = standardized root mean square residual. | | | | | |

**References**

1. . Schermelleh-Engel K, Moosbrugger H, Müller H. Evaluating the fit of structural equation models: Tests of significance and descriptive goodness-of-fit measures. Methods Psychol Res Online. 2003;8:23-74. [↑](#endnote-ref-1)
2. . Chen FF. Sensitivity of goodness of fit indexes to lack of measurement invariance. Struct Equ Modeling. 2007;14:464-504. [↑](#endnote-ref-2)
3. . Possible Questionnaire Format for Administering the 50-Item Set of IPIP Big-Five Factor Markers. (n.d.). Retrieved 21 March 2017, from http://ipip.ori.org/New_IPIP-50-item-scale.htm [↑](#endnote-ref-3)
