# Supplementary material for: Apolipoprotein E genotype does not moderate the associations of depressive symptoms, neuroticism and allostatic load with cognitive ability and cognitive aging in the Lothian Birth Cohort 1936
Source: PLoS One. 2018 Feb 16;13(2):e0192604. doi: 10.1371/journal.pone.0192604 (PMC5815580; doi:10.1371/journal.pone.0192604)
Supplement: S2 Appendix — Descriptive statistics for the groups of those with the APOE E3/E3 and E3/E4 genotypes, and correlations within all four groups. (DOCX) [file pone.0192604.s002.docx]

**Descriptive statistics and correlation matrices**

| **Table F. Descriptive Statistics for Raw Allostatic Load Biomarker Values in Groups of *APOE* E4 Non-carriers and Carriers.** | | | | | | | | | | | | | | | |
| --- | --- | --- | --- | --- | --- | --- | --- | --- | --- | --- | --- | --- | --- | --- | --- |
| **Variable** | ***n*** | | | ***M*** | | | ***SD*** | | | ***Skew*** | | | ***Kurtosis*** | | |
|  | **both groups** | **no E4 group** | **E4 group** | **both groups** | **no E4 group** | **E4 group** | **both groups** | **no E4 group** | **E4 group** | **both groups** | **no E4 group** | **E4 group** | **both groups** | **no E4 group** | **E4 group** |
| **Albumin** | 1014 | 712 | 302 | 44.66 | 44.63 | 44.72 | 3.08 | 3.18 | 2.86 | -0.03 | -0.07 | 0.10 | 0.89 | 0.96 | 0.46 |
| **CRP** | 1010 | 709 | 301 | 5.25 | 5.38 | 4.96 | 6.73 | 5.84 | 8.46 | 4.86 | 2.77 | 6.11 | 40.26 | 10.31 | 48.63 |
| **Log of CRP** | 1010 | 709 | 301 | 1.22 | 1.27 | 1.09 | 0.87 | 0.86 | 0.87 | 0.70 | 0.53 | 1.14 | -0.37 | -0.73 | 0.79 |
| **Fibrinogen** | 1008 | 708 | 300 | 3.28 | 3.30 | 3.23 | 0.64 | 0.63 | 0.64 | 0.63 | 0.46 | 1.02 | 1.07 | 0.45 | 2.67 |
| **Log of fibrinogen** | 1008 | 708 | 300 | 1.17 | 1.18 | 1.15 | 0.19 | 0.19 | 0.19 | -0.09 | -0.18 | 0.11 | 0.57 | 0.36 | 1.17 |
| **HbA1c** | 1017 | 713 | 304 | 5.94 | 5.95 | 5.89 | 0.77 | 0.79 | 0.70 | 3.02 | 2.86 | 3.46 | 16.84 | 15.56 | 20.57 |
| **Log of HbA1c** | 1017 | 713 | 304 | 1.77 | 1.78 | 1.77 | 0.11 | 0.12 | 0.10 | 1.57 | 1.39 | 2.13 | 9.34 | 8.96 | 10.05 |
| **HDLR** | 930 | 655 | 275 | 3.76 | 3.69 | 3.92 | 1.07 | 1.04 | 1.13 | 0.88 | 0.94 | 0.74 | 1.92 | 2.49 | 0.93 |
| **Triglyceride** | 925 | 651 | 274 | 1.65 | 1.64 | 1.67 | 0.92 | 0.79 | 1.17 | 5.49 | 2.12 | 7.41 | 71.12 | 8.62 | 86.34 |
| **Log of triglyceride** | 925 | 651 | 274 | 0.40 | 0.40 | 0.39 | 0.44 | 0.43 | 0.46 | 0.41 | 0.28 | 0.68 | 0.84 | 0.17 | 1.99 |
| **BMI** | 1027 | 722 | 305 | 27.81 | 27.79 | 27.84 | 4.36 | 4.35 | 4.40 | 0.83 | 0.79 | 0.91 | 1.77 | 1.73 | 1.83 |
| **Log of BMI** | 1027 | 722 | 305 | 3.31 | 3.31 | 3.31 | 0.15 | 0.15 | 0.15 | 0.18 | 0.14 | 0.30 | 0.75 | 0.77 | 0.67 |
| **SBP** | 1026 | 721 | 305 | 149.68 | 149.73 | 149.56 | 19.09 | 18.91 | 19.53 | 0.48 | 0.57 | 0.28 | 0.36 | 0.58 | -0.13 |
| **Log of SBP** | 1026 | 721 | 305 | 5.00 | 5.00 | 5.00 | 0.13 | 0.12 | 0.13 | 0.09 | 0.17 | -0.07 | 0.07 | 0.17 | -0.19 |
| **DBP** | 1026 | 721 | 305 | 81.36 | 81.89 | 80.12 | 10.21 | 10.43 | 9.57 | 0.30 | 0.41 | -0.10 | 0.44 | 0.49 | -0.13 |
| **Log of DBP** | 1026 | 721 | 305 | 4.39 | 4.40 | 4.38 | 0.13 | 0.13 | 0.12 | -0.11 | 0.01 | -0.46 | 0.14 | 0.02 | 0.22 |
| CRP = C-reactive protein. HbA1c = glycated haemoglobin. HDLR = high-density lipoprotein ratio. BMI = body mass index. SBP = systolic blood pressure. DBP = diastolic blood pressure. | | | | | | | | | | | | | | | |

| **Table G. Descriptive Statistics for Raw Allostatic Load Biomarker Values in Groups of Those with the *APOE* E3/E3 and E3/E4 Genotypes.** | | | | | | | | | | | | | | | |
| --- | --- | --- | --- | --- | --- | --- | --- | --- | --- | --- | --- | --- | --- | --- | --- |
| **Variable** | ***n*** | | | ***M*** | | | ***SD*** | | | ***Skew*** | | | ***Kurtosis*** | | |
|  | **both groups** | **E3/E3 group** | **E3/E4 group** | **both groups** | **E3/E3 group** | **E3/E4 group** | **both groups** | **E3/E3 group** | **E3/E4 group** | **both groups** | **E3/E3 group** | **E3/E4 group** | **both groups** | **E3/E3 group** | **E3/E4 group** |
| **Albumin** | 849 | 590 | 259 | 44.71 | 44.69 | 44.74 | 3.06 | 3.14 | 2.86 | 0.06 | 0.10 | -0.06 | 0.42 | 0.42 | 0.30 |
| **CRP** | 846 | 587 | 259 | 5.32 | 5.46 | 5.02 | 6.99 | 6.01 | 8.83 | 4.97 | 2.78 | 6.13 | 40.63 | 10.31 | 47.32 |
| **Log of CRP** | 846 | 587 | 259 | 1.22 | 1.28 | 1.09 | 0.88 | 0.87 | 0.88 | 0.71 | 0.53 | 1.15 | -0.36 | -0.75 | 0.86 |
| **Fibrinogen** | 844 | 586 | 258 | 3.28 | 3.30 | 3.24 | 0.64 | 0.64 | 0.64 | 0.69 | 0.46 | 1.24 | 1.25 | 0.57 | 2.97 |
| **Log of fibrinogen** | 844 | 586 | 258 | 1.17 | 1.18 | 1.16 | 0.19 | 0.19 | 0.19 | -0.03 | -0.23 | 0.46 | 0.54 | 0.52 | 0.73 |
| **HbA1c** | 852 | 591 | 261 | 5.93 | 5.96 | 5.88 | 0.75 | 0.77 | 0.69 | 2.60 | 2.24 | 3.64 | 11.65 | 8.37 | 22.68 |
| **Log of HbA1c** | 852 | 591 | 261 | 1.77 | 1.78 | 1.77 | 0.11 | 0.12 | 0.10 | 1.36 | 1.08 | 2.24 | 8.43 | 7.63 | 10.97 |
| **HDLR** | 775 | 538 | 237 | 3.79 | 3.73 | 3.94 | 1.09 | 1.05 | 1.15 | 0.90 | 0.97 | 0.73 | 2.01 | 2.67 | 0.94 |
| **Triglyceride** | 772 | 535 | 237 | 1.61 | 1.62 | 1.61 | 0.75 | 0.75 | 0.74 | 1.58 | 1.68 | 1.36 | 3.82 | 4.68 | 1.72 |
| **Log of triglyceride** | 772 | 535 | 237 | 0.39 | 0.39 | 0.38 | 0.42 | 0.43 | 0.42 | 0.22 | 0.20 | 0.27 | -0.04 | -0.01 | -0.12 |
| **BMI** | 858 | 597 | 261 | 27.81 | 27.75 | 27.97 | 4.46 | 4.45 | 4.49 | 0.79 | 0.74 | 0.90 | 1.68 | 1.59 | 1.82 |
| **Log of BMI** | 858 | 597 | 261 | 3.31 | 3.31 | 3.32 | 0.16 | 0.16 | 0.16 | 0.14 | 0.09 | 0.26 | 0.70 | 0.68 | 0.70 |
| **SBP** | 857 | 596 | 261 | 150.06 | 149.73 | 150.83 | 18.91 | 18.49 | 19.83 | 0.51 | 0.63 | 0.26 | 0.43 | 0.78 | -0.20 |
| **Log of SBP** | 857 | 596 | 261 | 5.00 | 5.00 | 5.01 | 0.12 | 0.12 | 0.13 | 0.11 | 0.22 | -0.09 | 0.11 | 0.28 | -0.21 |
| **DBP** | 857 | 596 | 261 | 81.61 | 82.14 | 80.39 | 10.15 | 10.28 | 9.75 | 0.22 | 0.34 | -0.13 | 0.42 | 0.52 | -0.17 |
| **Log of DBP** | 857 | 596 | 261 | 4.39 | 4.40 | 4.38 | 0.13 | 0.13 | 0.12 | -0.19 | -0.07 | -0.48 | 0.20 | 0.13 | 0.16 |
| CRP = C-reactive protein. HbA1c = glycated haemoglobin. HDLR = high-density lipoprotein ratio. BMI = body mass index. SBP = systolic blood pressure. DBP = diastolic blood pressure. | | | | | | | | | | | | | | | |

| **Table H. Descriptive Statistics for Groups of Those with the *APOE* E3/E3 and E3/E4 Genotypes.** | | | | | | | | | | | | | | | | |
| --- | --- | --- | --- | --- | --- | --- | --- | --- | --- | --- | --- | --- | --- | --- | --- | --- |
| **Variable** | | ***n*** | | | ***M*** | | | ***SD*** | | | ***Skew*** | | | ***Kurtosis*** | | |
|  |  | **both groups** | **E3/E3 group** | **E3/E4 group** | **both groups** | **E3/E3 group** | **E3/E4 group** | **both groups** | **E3/E3 group** | **E3/E4 group** | **both groups** | **E3/E3 group** | **E3/E4 group** | **both groups** | **E3/E3 group** | **E3/E4 group** |
| **Block Design** | **W1** | 855 | 594 | 261 | 33.95 | 34.08 | 33.67 | 10.31 | 10.29 | 10.39 | 0.30 | 0.32 | 0.25 | -0.27 | -0.18 | -0.50 |
|  | **W2** | 683 | 474 | 209 | 33.83 | 34.14 | 33.11 | 10.05 | 10.26 | 9.54 | 0.51 | 0.45 | 0.64 | 0.12 | 0.09 | 0.16 |
|  | **W3** | 545 | 374 | 171 | 32.35 | 32.92 | 31.12 | 10.08 | 9.93 | 10.31 | 0.34 | 0.33 | 0.39 | 0.28 | 0.29 | 0.28 |
| **Digit Symbol** | **W1** | 854 | 592 | 262 | 56.69 | 56.94 | 56.11 | 12.89 | 13.16 | 12.26 | 0.03 | 0.01 | 0.07 | -0.21 | -0.23 | -0.21 |
|  | **W2** | 682 | 473 | 209 | 56.39 | 56.55 | 56.03 | 12.35 | 12.69 | 11.58 | 0.04 | 0.03 | 0.05 | -0.22 | -0.44 | 0.40 |
|  | **W3** | 538 | 370 | 168 | 53.62 | 54.82 | 50.96 | 12.95 | 12.37 | 13.82 | -0.08 | -0.10 | 0.07 | -0.15 | -0.13 | -0.22 |
| **Letter-Number Sequencing** | **W1** | 850 | 591 | 259 | 10.96 | 11.08 | 10.71 | 3.13 | 3.18 | 3.00 | 0.15 | 0.14 | 0.14 | 0.04 | -0.05 | 0.22 |
|  | **W2** | 684 | 475 | 209 | 10.91 | 10.92 | 10.89 | 2.95 | 3.08 | 2.64 | 0.18 | 0.18 | 0.17 | 0.34 | 0.19 | 0.65 |
|  | **W3** | 543 | 375 | 168 | 10.41 | 10.52 | 10.15 | 2.86 | 2.87 | 2.83 | 0.03 | 0.07 | -0.08 | 0.16 | 0.24 | -0.11 |
| **Matrix Reasoning** | **W1** | 856 | 595 | 261 | 13.46 | 13.69 | 12.95 | 5.11 | 5.19 | 4.90 | 0.01 | -0.03 | 0.08 | -0.97 | -0.96 | -1.00 |
|  | **W2** | 683 | 474 | 209 | 13.16 | 13.31 | 12.83 | 5.01 | 5.08 | 4.83 | 0.00 | -0.05 | 0.10 | -1.05 | -1.03 | -1.11 |
|  | **W3** | 543 | 374 | 169 | 12.96 | 13.26 | 12.28 | 4.92 | 4.93 | 4.84 | 0.05 | 0.02 | 0.11 | -0.94 | -0.95 | -0.97 |
| **Symbol Search** | **W1** | 856 | 594 | 262 | 24.79 | 25.07 | 24.16 | 6.37 | 6.23 | 6.64 | 0.00 | -0.02 | 0.07 | 0.68 | 0.11 | 1.66 |
|  | **W2** | 682 | 473 | 209 | 24.64 | 24.80 | 24.29 | 6.17 | 6.15 | 6.20 | -0.29 | -0.29 | -0.28 | 0.84 | 0.79 | 0.93 |
|  | **W3** | 541 | 373 | 168 | 24.50 | 25.41 | 22.48 | 6.51 | 6.19 | 6.74 | -0.15 | -0.17 | 0.01 | 0.88 | 0.30 | 2.05 |
| **Age** | **W1** | 859 | 597 | 262 | 69.52 | 2.50 | 2.55 | 0.82 | 0.82 | 0.83 | -0.03 | -0.05 | 0.00 | -0.88 | -0.86 | -0.94 |
|  | **W2** | 685 | 476 | 209 | 72.47 | 5.46 | 5.49 | 0.70 | 0.69 | 0.72 | -0.03 | -0.06 | 0.01 | -0.85 | -0.79 | -1.01 |
|  | **W3** | 550 | 379 | 171 | 76.23 | 9.20 | 9.31 | 0.67 | 0.66 | 0.67 | -0.06 | -0.05 | -0.09 | -0.89 | -0.79 | -1.15 |
| **Number of medical conditions** | **W1** | 852 | 594 | 258 | 2.91 | 2.93 | 2.87 | 1.63 | 1.67 | 1.52 | 0.48 | 0.56 | 0.21 | 0.13 | 0.26 | -0.51 |
|  | **W2** | 685 | 476 | 209 | 3.39 | 3.33 | 3.51 | 1.72 | 1.73 | 1.70 | 0.35 | 0.35 | 0.36 | -0.24 | -0.32 | -0.05 |
|  | **W3** | 544 | 374 | 170 | 3.90 | 3.90 | 3.92 | 1.73 | 1.78 | 1.63 | 0.38 | 0.27 | 0.71 | 0.17 | -0.11 | 0.87 |
| **Dep. symptoms** | **W1** | 855 | 594 | 261 | 2.76 | 2.83 | 2.61 | 2.20 | 2.19 | 2.22 | 1.42 | 1.23 | 1.86 | 2.82 | 1.55 | 5.77 |
| **Neuroticism parcel** | **1** | 758 | 525 | 233 | 2.13 | 2.13 | 2.14 | 0.83 | 0.81 | 0.89 | 0.60 | 0.52 | 0.72 | -0.14 | -0.25 | -0.06 |
|  | **2** | 757 | 525 | 232 | 2.61 | 2.62 | 2.59 | 0.82 | 0.79 | 0.87 | 0.22 | 0.16 | 0.32 | -0.22 | -0.32 | -0.13 |
|  | **3** | 758 | 525 | 233 | 2.43 | 2.44 | 2.41 | 0.79 | 0.78 | 0.80 | 0.17 | 0.11 | 0.29 | -0.32 | -0.35 | -0.27 |
| **Allostatic load** | **W1** | 855 | 594 | 261 | 0.74 | 0.74 | 0.74 | 0.25 | 0.24 | 0.26 | 0.88 | 0.76 | 1.08 | 1.07 | 0.69 | 1.65 |
| **Neuroticism facet parcels** | **Dep** | 758 | 525 | 233 | 2.21 | 2.20 | 2.24 | 0.89 | 0.88 | 0.93 | 0.46 | 0.38 | 0.61 | -0.40 | -0.62 | -0.09 |
|  | **Anx** | 758 | 525 | 233 | 2.72 | 2.73 | 2.70 | 0.86 | 0.84 | 0.91 | 0.11 | 0.12 | 0.11 | -0.42 | -0.43 | -0.46 |
|  | **Ang** | 637 | 436 | 201 | 2.49 | 2.52 | 2.42 | 0.87 | 0.87 | 0.86 | 0.22 | 0.11 | 0.46 | -0.74 | -0.88 | -0.34 |
|  | | **Females** | | | **Males** | | |  | | | | | | | | |
|  |  | 425 | 304 | 121 | 434 | 293 | 141 |  |  |  |  |  |  |  |  |  |
| W1 = Wave 1. W2 = Wave 2. W3 = Wave 3. Dep. symptoms = depressive symptoms. Dep = depression. Anx = anxiety. Ang = anger. | | | | | | | | | | | | | | | | |

| **Table I. Pearson's Correlation Matrix for All Primary Analysis Model Variables in Groups of *APOE* E4 Non-carriers and Carriers.** | | | | | | | | | | | | | | | | | | | | | | | | | | | | |
| --- | --- | --- | --- | --- | --- | --- | --- | --- | --- | --- | --- | --- | --- | --- | --- | --- | --- | --- | --- | --- | --- | --- | --- | --- | --- | --- | --- | --- |
| **Variable** | | **BD** | | | **DS** | | | **LN** | | | **MR** | | | **SS** | | | **Age** | | | **MC** | | | **D** | **NP** | | | **AL** | **Sex** |
|  |  | **W1** | **W2** | **W3** | **W1** | **W2** | **W3** | **W1** | **W2** | **W3** | **W1** | **W2** | **W3** | **W1** | **W2** | **W3** | **W1** | **W2** | **W3** | **W1** | **W2** | **W3** | **W1** | **1** | **2** | **3** | **W1** |  |
| **BD** | **W1** |  | .79 | .73 | .36 | .36 | .32 | .41 | .28 | .30 | .59 | .51 | .59 | .49 | .46 | .41 | -.10 | -.05 | -.01 | -.17 | -.15 | -.22 | -.15 | -.20 | -.24 | -.21 | -.03 | .18 |
|  | **W2** | .75 |  | .75 | .36 | .39 | .32 | .35 | .28 | .31 | .53 | .54 | .54 | .42 | .49 | .43 | -.05 | -.06 | -.03 | -.20 | -.16 | -.18 | -.13 | -.21 | -.28 | -.22 | -.06 | .14 |
|  | **W3** | .78 | .76 |  | .37 | .43 | .45 | .39 | .31 | .41 | .54 | .59 | .62 | .49 | .49 | .56 | .02 | .04 | .02 | -.16 | -.20 | -.21 | -.10 | -.16 | -.19 | -.10 | -.05 | .08 |
| **DS** | **W1** | .41 | .39 | .37 |  | .83 | .69 | .40 | .39 | .33 | .29 | .36 | .31 | .56 | .52 | .51 | -.13 | -.12 | -.17 | -.19 | -.17 | -.19 | -.21 | -.09 | -.04 | -.08 | -.17 | -.17 |
|  | **W2** | .42 | .41 | .39 | .85 |  | .78 | .36 | .41 | .40 | .29 | .38 | .35 | .53 | .58 | .57 | -.15 | -.14 | -.18 | -.25 | -.22 | -.20 | -.28 | -.07 | -.06 | -.11 | -.21 | -.16 |
|  | **W3** | .40 | .37 | .43 | .81 | .86 |  | .29 | .30 | .38 | .26 | .37 | .39 | .45 | .49 | .65 | -.13 | -.09 | -.12 | -.21 | -.23 | -.18 | -.19 | -.15 | -.08 | -.09 | -.20 | -.16 |
| **LN** | **W1** | .39 | .39 | .39 | .41 | .42 | .40 |  | .59 | .51 | .36 | .29 | .33 | .48 | .43 | .31 | -.12 | -.08 | -.10 | -.16 | -.08 | -.16 | -.09 | -.13 | -.15 | -.14 | -.03 | .00 |
|  | **W2** | .38 | .39 | .35 | .40 | .43 | .45 | .63 |  | .66 | .26 | .31 | .30 | .36 | .40 | .33 | .05 | .05 | .07 | -.03 | .03 | .01 | -.04 | -.09 | -.08 | -.09 | -.09 | -.07 |
|  | **W3** | .29 | .25 | .28 | .37 | .41 | .43 | .56 | .68 |  | .34 | .37 | .38 | .34 | .32 | .38 | .01 | .02 | .02 | .00 | -.03 | -.02 | -.05 | -.13 | -.09 | -.05 | -.02 | -.09 |
| **MR** | **W1** | .56 | .51 | .54 | .40 | .39 | .37 | .46 | .41 | .34 |  | .60 | .66 | .43 | .39 | .31 | -.10 | -.10 | -.08 | -.08 | -.06 | -.20 | -.14 | -.13 | -.19 | -.17 | .00 | .09 |
|  | **W2** | .57 | .53 | .51 | .36 | .38 | .37 | .36 | .40 | .28 | .66 |  | .62 | .41 | .44 | .43 | -.02 | -.03 | -.04 | -.17 | -.13 | -.20 | -.08 | -.14 | -.15 | -.20 | -.07 | .12 |
|  | **W3** | .55 | .50 | .53 | .36 | .37 | .40 | .38 | .38 | .36 | .62 | .65 |  | .35 | .38 | .42 | .03 | .06 | .03 | -.14 | -.19 | -.19 | -.21 | -.26 | -.22 | -.20 | .02 | .05 |
| **SS** | **W1** | .47 | .48 | .50 | .65 | .63 | .62 | .44 | .39 | .32 | .45 | .36 | .39 |  | .65 | .54 | -.24 | -.20 | -.23 | -.17 | -.17 | -.23 | -.14 | -.17 | -.18 | -.17 | -.05 | -.08 |
|  | **W2** | .48 | .48 | .51 | .62 | .65 | .62 | .37 | .40 | .31 | .40 | .36 | .36 | .69 |  | .63 | -.10 | -.10 | -.10 | -.26 | -.23 | -.24 | -.12 | -.08 | -.15 | -.14 | -.11 | .03 |
|  | **W3** | .46 | .47 | .51 | .61 | .65 | .66 | .41 | .42 | .40 | .39 | .38 | .43 | .67 | .68 |  | -.13 | -.10 | -.12 | -.13 | -.20 | -.14 | -.10 | -.16 | -.14 | -.14 | -.03 | -.01 |
| **Age** | **W1** | -.16 | -.18 | -.16 | -.21 | -.18 | -.17 | -.21 | -.12 | -.14 | -.17 | -.15 | -.08 | -.24 | -.12 | -.15 |  | .96 | .96 | .17 | .11 | .12 | .06 | .06 | .05 | .10 | .00 | .00 |
|  | **W2** | -.16 | -.17 | -.14 | -.18 | -.17 | -.15 | -.20 | -.09 | -.14 | -.16 | -.12 | -.05 | -.23 | -.12 | -.14 | .94 |  | .95 | .21 | .12 | .12 | .03 | .09 | .08 | .09 | .04 | -.01 |
|  | **W3** | -.17 | -.17 | -.14 | -.19 | -.19 | -.18 | -.21 | -.12 | -.11 | -.12 | -.08 | -.06 | -.21 | -.11 | -.16 | .93 | .91 |  | .18 | .11 | .13 | .04 | .05 | .04 | .04 | .07 | .03 |
| **MC** | **W1** | -.09 | -.12 | -.09 | -.18 | -.17 | -.20 | -.18 | -.13 | -.07 | -.11 | -.11 | -.07 | -.13 | -.10 | -.13 | .12 | .11 | .11 |  | .72 | .68 | .18 | .13 | .17 | .16 | .09 | -.08 |
|  | **W2** | -.05 | -.09 | -.09 | -.15 | -.15 | -.17 | -.10 | -.10 | -.10 | -.07 | -.08 | -.03 | -.11 | -.05 | -.08 | .13 | .13 | .12 | .78 |  | .71 | .12 | .10 | .11 | .08 | .18 | -.06 |
|  | **W3** | -.07 | -.12 | -.09 | -.14 | -.15 | -.20 | -.11 | -.13 | -.12 | -.05 | -.06 | -.05 | -.16 | -.14 | -.12 | .11 | .11 | .12 | .64 | .78 |  | .20 | .05 | .10 | .10 | .16 | -.07 |
| **D** | **W1** | -.07 | -.07 | -.13 | -.16 | -.15 | -.15 | -.10 | -.10 | -.06 | -.08 | -.05 | -.10 | -.14 | -.11 | -.17 | .02 | .02 | .07 | .20 | .16 | .19 |  | .28 | .31 | .32 | .05 | .04 |
| **NP** | **1** | -.17 | -.20 | -.18 | -.15 | -.15 | -.20 | -.19 | -.19 | -.07 | -.10 | -.17 | -.21 | -.15 | -.16 | -.18 | .02 | .01 | .00 | .21 | .15 | .14 | .32 |  | .79 | .68 | .05 | -.06 |
|  | **2** | -.18 | -.20 | -.17 | -.12 | -.10 | -.13 | -.17 | -.15 | -.05 | -.08 | -.15 | -.17 | -.12 | -.14 | -.12 | .01 | .03 | -.03 | .21 | .19 | .15 | .31 | .75 |  | .71 | .07 | -.17 |
|  | **3** | -.13 | -.16 | -.18 | -.16 | -.09 | -.14 | -.12 | -.13 | -.05 | -.07 | -.14 | -.16 | -.12 | -.13 | -.09 | .04 | .04 | .01 | .17 | .14 | .09 | .29 | .61 | .61 |  | .07 | -.07 |
| **AL** | **W1** | -.07 | -.09 | -.12 | -.12 | -.16 | -.16 | -.04 | -.03 | -.06 | -.08 | -.10 | -.10 | -.09 | -.07 | -.10 | .06 | .04 | .03 | .16 | .20 | .16 | .16 | .11 | .04 | .04 |  | .10 |
| **Sex** | | .15 | .15 | .16 | -.15 | -.17 | -.14 | .01 | .02 | .01 | .10 | .08 | .06 | .02 | -.02 | -.01 | -.01 | -.03 | .02 | -.05 | -.01 | -.03 | .05 | -.17 | -.14 | -.01 | -.02 |  |
| Correlations for the groups of *APOE* E4 non-carriers and carriers are on the lower and upper triangles of the matrix respectively. For sex, females were the reference group. BD = Block Design. DS = Digit Symbol Coding. LN = Letter-Number Sequencing. MR = Matrix Reasoning. SS = Symbol Search. MC = medical conditions. D = depressive symptoms. NP = neuroticism parcel. AL = allostatic load. W1 = Wave 2. W2 = Wave 2. W3 = Wave 3. | | | | | | | | | | | | | | | | | | | | | | | | | | | | |

| **Table J. Pearson's Correlation Matrix for All Primary Analysis Model Variables in Groups of Those with the *APOE* E3/E3 and E3/E4 Genotypes.** | | | | | | | | | | | | | | | | | | | | | | | | | | | | |
| --- | --- | --- | --- | --- | --- | --- | --- | --- | --- | --- | --- | --- | --- | --- | --- | --- | --- | --- | --- | --- | --- | --- | --- | --- | --- | --- | --- | --- |
| **Variable** | | **BD** | | | **DS** | | | **LN** | | | **MR** | | | **SS** | | | **Age** | | | **MC** | | | **D** | **NP** | | | **AL** | **Sex** |
|  |  | **W1** | **W2** | **W3** | **W1** | **W2** | **W3** | **W1** | **W2** | **W3** | **W1** | **W2** | **W3** | **W1** | **W2** | **W3** | **W1** | **W2** | **W3** | **W1** | **W2** | **W3** | **W1** | **1** | **2** | **3** | **W1** |  |
| **BD** | **W1** |  | .80 | .71 | .32 | .32 | .28 | .38 | .27 | .32 | .60 | .50 | .61 | .46 | .42 | .39 | -.10 | -.02 | .01 | -.15 | -.11 | -.22 | -.15 | -.22 | -.22 | -.20 | .01 | .18 |
|  | **W2** | .75 |  | .78 | .35 | .39 | .31 | .36 | .30 | .34 | .53 | .52 | .56 | .43 | .47 | .46 | -.04 | -.04 | -.02 | -.18 | -.15 | -.20 | -.09 | -.23 | -.27 | -.22 | -.04 | .17 |
|  | **W3** | .78 | .76 |  | .32 | .39 | .41 | .34 | .32 | .41 | .55 | .60 | .64 | .45 | .47 | .55 | .05 | .07 | .06 | -.17 | -.21 | -.23 | -.10 | -.20 | -.20 | -.16 | -.04 | .10 |
| **DS** | **W1** | .42 | .37 | .37 |  | .83 | .66 | .40 | .33 | .26 | .24 | .33 | .31 | .52 | .50 | .48 | -.14 | -.10 | -.17 | -.18 | -.17 | -.14 | -.22 | -.11 | -.01 | -.07 | -.18 | -.17 |
|  | **W2** | .43 | .39 | .40 | .86 |  | .79 | .34 | .34 | .37 | .26 | .34 | .34 | .48 | .56 | .57 | -.16 | -.15 | -.22 | -.24 | -.23 | -.19 | -.26 | -.06 | -.03 | -.06 | -.22 | -.18 |
|  | **W3** | .42 | .36 | .46 | .82 | .85 |  | .23 | .27 | .34 | .21 | .33 | .39 | .39 | .47 | .63 | -.09 | -.06 | -.08 | -.22 | -.25 | -.16 | -.19 | -.16 | -.06 | -.10 | -.20 | -.15 |
| **LN** | **W1** | .41 | .39 | .41 | .40 | .41 | .38 |  | .59 | .49 | .32 | .24 | .28 | .46 | .36 | .23 | -.10 | -.05 | -.07 | -.15 | -.05 | -.12 | -.05 | -.12 | -.11 | -.11 | -.05 | -.03 |
|  | **W2** | .40 | .41 | .38 | .42 | .43 | .46 | .62 |  | .65 | .16 | .25 | .24 | .33 | .34 | .30 | .01 | .01 | .00 | -.04 | .02 | .04 | .00 | -.08 | -.05 | -.04 | -.15 | -.05 |
|  | **W3** | .30 | .27 | .31 | .40 | .41 | .45 | .54 | .65 |  | .28 | .35 | .33 | .31 | .30 | .34 | .02 | .03 | .02 | -.05 | -.06 | -.02 | -.03 | -.15 | -.07 | -.07 | -.09 | -.04 |
| **MR** | **W1** | .56 | .50 | .52 | .38 | .38 | .36 | .46 | .43 | .35 |  | .57 | .63 | .39 | .33 | .27 | -.12 | -.10 | -.08 | -.06 | -.04 | -.18 | -.14 | -.13 | -.15 | -.18 | .01 | .11 |
|  | **W2** | .57 | .53 | .52 | .36 | .39 | .38 | .39 | .43 | .31 | .67 |  | .62 | .37 | .39 | .41 | -.02 | -.02 | -.04 | -.17 | -.14 | -.20 | -.05 | -.10 | -.10 | -.18 | -.06 | .13 |
|  | **W3** | .58 | .50 | .54 | .38 | .37 | .40 | .38 | .37 | .37 | .61 | .66 |  | .35 | .37 | .44 | .01 | .05 | .01 | -.17 | -.21 | -.22 | -.22 | -.27 | -.17 | -.24 | .00 | .04 |
| **SS** | **W1** | .47 | .47 | .50 | .65 | .64 | .64 | .45 | .43 | .35 | .42 | .35 | .37 |  | .65 | .52 | -.26 | -.20 | -.22 | -.18 | -.19 | -.22 | -.13 | -.20 | -.17 | -.17 | -.03 | -.08 |
|  | **W2** | .46 | .47 | .50 | .62 | .65 | .62 | .37 | .42 | .32 | .37 | .35 | .34 | .69 |  | .63 | -.10 | -.09 | -.09 | -.22 | -.23 | -.20 | -.12 | -.06 | -.13 | -.12 | -.11 | .04 |
|  | **W3** | .46 | .47 | .52 | .60 | .65 | .66 | .39 | .42 | .41 | .37 | .38 | .43 | .68 | .66 |  | -.08 | -.07 | -.08 | -.16 | -.23 | -.15 | -.12 | -.17 | -.15 | -.16 | -.04 | .02 |
| **Age** | **W1** | -.18 | -.21 | -.19 | -.22 | -.21 | -.19 | -.20 | -.13 | -.16 | -.18 | -.17 | -.10 | -.25 | -.15 | -.17 |  | .96 | .96 | .16 | .08 | .09 | .07 | .06 | .05 | .10 | -.04 | .01 |
|  | **W2** | -.20 | -.19 | -.18 | -.20 | -.19 | -.17 | -.19 | -.10 | -.15 | -.17 | -.15 | -.09 | -.25 | -.15 | -.17 | .95 |  | .95 | .18 | .06 | .05 | .04 | .09 | .10 | .09 | -.01 | .01 |
|  | **W3** | -.20 | -.19 | -.17 | -.20 | -.20 | -.20 | -.21 | -.12 | -.13 | -.13 | -.11 | -.09 | -.22 | -.13 | -.18 | .93 | .92 |  | .19 | .11 | .09 | .08 | .08 | .06 | .07 | .04 | .03 |
| **MC** | **W1** | -.09 | -.11 | -.11 | -.19 | -.17 | -.18 | -.18 | -.11 | -.05 | -.09 | -.08 | -.06 | -.13 | -.10 | -.14 | .16 | .16 | .20 |  | .68 | .65 | .20 | .14 | .16 | .13 | .04 | -.03 |
|  | **W2** | -.05 | -.08 | -.11 | -.18 | -.17 | -.18 | -.12 | -.11 | -.09 | -.09 | -.08 | -.05 | -.12 | -.06 | -.09 | .17 | .18 | .17 | .79 |  | .69 | .16 | .10 | .09 | .05 | .12 | -.03 |
|  | **W3** | -.05 | -.11 | -.12 | -.15 | -.13 | -.17 | -.11 | -.11 | -.09 | -.07 | -.04 | -.06 | -.18 | -.14 | -.13 | .16 | .16 | .15 | .66 | .77 |  | .21 | .05 | .07 | .09 | .08 | -.05 |
| **D** | **W1** | -.08 | -.07 | -.15 | -.18 | -.15 | -.16 | -.10 | -.10 | -.05 | -.05 | -.03 | -.09 | -.15 | -.12 | -.18 | .06 | .05 | .12 | .21 | .19 | .22 |  | .28 | .27 | .29 | .05 | .04 |
| **NP** | **1** | -.17 | -.20 | -.17 | -.16 | -.14 | -.20 | -.19 | -.17 | -.08 | -.10 | -.17 | -.22 | -.14 | -.18 | -.17 | .03 | .00 | .01 | .24 | .17 | .16 | .30 |  | .79 | .69 | .04 | -.01 |
|  | **2** | -.17 | -.19 | -.15 | -.11 | -.09 | -.13 | -.17 | -.14 | -.02 | -.06 | -.16 | -.17 | -.09 | -.16 | -.09 | -.01 | -.01 | -.05 | .23 | .20 | .18 | .30 | .75 |  | .71 | .07 | -.15 |
|  | **3** | -.12 | -.14 | -.16 | -.14 | -.05 | -.12 | -.10 | -.10 | -.01 | -.05 | -.15 | -.14 | -.08 | -.11 | -.07 | .04 | .03 | .03 | .19 | .15 | .08 | .32 | .65 | .64 |  | .08 | -.04 |
| **AL** | **W1** | -.10 | -.10 | -.15 | -.13 | -.19 | -.17 | -.07 | -.04 | -.09 | -.10 | -.11 | -.11 | -.10 | -.11 | -.12 | .07 | .04 | .04 | .17 | .23 | .20 | .14 | .10 | .02 | .05 |  | .14 |
| **Sex** | | .15 | .17 | .15 | -.14 | -.16 | -.12 | .01 | .01 | .00 | .10 | .07 | .05 | .02 | -.03 | .00 | -.04 | -.05 | -.02 | -.03 | .00 | -.04 | .05 | -.17 | -.14 | -.02 | -.03 |  |
| Correlations for the groups of those with the *APOE* E3/E3 and E3/E4 genotypes are on the lower and upper triangles of the matrix respectively. For sex, females were the reference group. BD = Block Design. DS = Digit Symbol Coding. LN = Letter-Number Sequencing. MR = Matrix Reasoning. SS = Symbol Search. MC = medical conditions. D = depressive symptoms. NP = neuroticism parcel. AL = allostatic load. W1 = Wave 2. W2 = Wave 2. W3 = Wave 3. | | | | | | | | | | | | | | | | | | | | | | | | | | | | |
