# Supplementary material for: Apolipoprotein E genotype does not moderate the associations of depressive symptoms, neuroticism and allostatic load with cognitive ability and cognitive aging in the Lothian Birth Cohort 1936
Source: PLoS One. 2018 Feb 16;13(2):e0192604. doi: 10.1371/journal.pone.0192604 (PMC5815580; doi:10.1371/journal.pone.0192604)
Supplement: S3 Appendix — Main and moderation effect test results, including for mediation models, in groups of those with the APOE E3/E3 and E3/E4 genotypes. (DOCX) [file pone.0192604.s003.docx]

**Results in groups of those with the *APOE* E3/E3 and E3/E4 genotypes**

The main, moderation and mediation effect specific genotypes results are in Tables K-M. Results were similar across groups, with a few differences to be noted here. Unlike in the E4 status grouping, there was no statistically significant main effect of *APOE* genotype on cognitive ability. Also, unlike in the other three groups, the estimates for the effects of depressive symptoms and allostatic load in the E3/E4 group were not statistically significant. Plus, in the mediation models, there was a significant effect of AL on the slope in the E3/E3 group.

| **Table K. Main Effect and Moderation Tests in Groups of Those with the *APOE* E3/E3 and E3/E4 Genotypes.** | | | | | | | | | | |
| --- | --- | --- | --- | --- | --- | --- | --- | --- | --- | --- |
| **Predictor** | **Regression constrained** | **Regression of intercept on predictor *estimate* (*SE*)** | | **Regression of slope on predictor *estimate* (*SE*)** | | **χ^2^_SB_ (Δχ^2^_SB_)** | ***df* (Δχ^2^_SB_ *df*)** | **Δχ^2^_SB_ *p*** | ***RMSEA*** | ***SRMR*** |
|  |  | **E3/E3 group** | **E3/E4 group** | **E3/E3 group** | **E3/E4 group** |  |  |  |  |  |
| **Depressive symptoms** | **None** | -0.17  (0.05)*** | -0.19  (0.08)* | -0.91  (0.26)*** | -0.79  (0.73) | 787.22 (N/A) | 380 (N/A) | N/A | .050 | .073 |
| **Depressive symptoms** | **Intercept** | -0.17  (0.04)*** | -0.19  (0.05)*** | -0.91  (0.26)*** | -0.80  (0.71) | 787.16 (0.01) | 381 (1) | .93 | .050 | .073 |
| **Depressive symptoms** | **Slope** | -0.17  (0.05)*** | -0.19  (0.08)* | -0.90  (0.26)*** | -0.82  (0.63) | 787.82 (0.01) | 381 (1) | .93 | .050 | .073 |
| **Neuroticism** | **None** | -0.23  (0.05)*** | -0.23  (0.08)** | -0.81  (0.53) | -0.59  (1.88) | 915.38 (N/A) | 472 | N/A | .047 | .069 |
| **Neuroticism** | **Intercept** | -0.22  (0.04)*** | -0.26  (0.05)*** | -0.83  (0.47) | -0.41  (2.56) | 915.44 (0.20) | 473 (1) | .66 | .047 | .069 |
| **Neuroticism** | **Slope** | -0.23  (0.05)*** | -0.23  (0.08)** | -0.77  (0.61) | -0.78  (0.92) | 914.77 (0.03) | 473 (1) | .87 | .047 | .069 |
| **Allostatic load** | **None** | -0.12  (0.05)** | -0.09  (0.07) | -0.84  (0.40)* | -0.96  (0.34)** | 788.27 (N/A) | 380 (N/A) | N/A | .050 | .075 |
| **Allostatic load** | **Intercept** | -0.11  (0.04)** | -0.12  (0.04)** | -0.86  (0.37)* | -0.94  (0.43)* | 788.46 (0.27) | 381 (1) | .60 | .050 | .075 |
| **Allostatic load** | **Slope** | -0.12  (0.05)** | -0.01  (0.08) | -0.87  (0.32)** | -0.91  (0.55) | 787.83 (0.06) | 381 (1) | .80 | .050 | .075 |
| *N* = 859. E3/E3 group *n* = 597. E3/E4 group *n* = 262. All χ^2^_SB_ *p*s < .001. All estimates are standardized. _SB_ = Satorra-Bentler. *RMSEA* = root mean square error of approximation. *SRMR* = standardized root mean square residual.  **p* ≤ .05. ***p* ≤ .01. ****p* ≤ .001. | | | | | | | | | | |

| **Table L. Parameter Estimates from the Mediation Model in Groups of Those with the *APOE* E3/E3 and E3/E4 Genotypes.** | | | | | | | | | | |
| --- | --- | --- | --- | --- | --- | --- | --- | --- | --- | --- |
| **Regression constrained to equality** | **Intercept on N *est.* (*SE*)** | | **Slope on N *est.* (*SE*)** | | **AL on N *est.* (*SE*)** | | **Intercept on AL *est.* (*SE*)** | | **Slope on AL *est.* (*SE*)** | |
|  | **E3/E3 group** | **E3/E4 group** | **E3/E3 group** | **E3/E4 group** | **E3/E3 group** | **E3/E4 group** | **E3/E3 group** | **E3/E4 group** | **E3/E3 group** | **E3/E4 group** |
| **None** | -0.22  (0.05)*** | -0.22  (0.08)** | -0.51  (0.54) | -0.19  (1.07) | 0.06  (0.05) | 0.08  (0.08) | -0.11  (0.05)* | -0.07  (0.07) | -0.71  (0.44) | -0.87  (0.51) |
| **Intercept on AL** | -0.23  (0.05)*** | -0.22  (0.09)** | -0.49  (0.52) | -0.23  (1.19) | 0.06  (0.05) | 0.08  (0.08) | -0.09  (0.04)* | -0.11  (0.04)* | -0.74  (0.42) | -0.84  (0.63) |
| **Slope on AL** | -0.22  (0.05)*** | -0.22  (0.09)** | -0.48  (0.51) | -0.27  (1.31) | 0.06  (0.05) | 0.08  (0.08) | -0.11  (0.05)* | -0.08  (0.08) | -0.75  (0.37)* | -0.81  (0.74) |
| *N* = 859. E3/E3 group *n* = 597. E3/E4 group *n* = 262. All estimates are standardized. N = neuroticism. AL = allostatic load.  **p* ≤ .05. ***p* ≤ .01. ****p* ≤ .001. | | | | | | | | | | |

| **Table M. Tests of Mediation and Moderated Mediation in Groups of Those with the *APOE* E3/E3 and E3/E4 Genotypes.** | | | | | | | | | |
| --- | --- | --- | --- | --- | --- | --- | --- | --- | --- |
| **Regression constrained to equality** | **Indirect effect on intercept *est.* (*SE*)** | | **Indirect effect on slope *est.* (*SE*)** | | **χ^2^_SB_ (Δχ^2^_SB_)** | ***df* (Δχ^2^_SB_ *df*)** | **Δχ^2^_SB_ *p*** | ***RMSEA*** | ***SRMR*** |
|  | **E3/E3 group** | **E3/E4 group** | **E3/E3 group** | **E3/E4 group** |  |  |  |  |  |
| **None** | -0.007 (0.006) | -0.006 (0.008) | -0.043 (0.044) | -0.071 (0.082) | 962.97 (N/A) | 508 (N/A) | N/A | .046 | .066 |
| **Intercept on AL** | -0.006 (0.005) | -0.009 (0.009) | -0.045 (0.043) | -0.067 (0.085) | 963.30 (0.40) | 509 (1) | .53 | .046 | .067 |
| **Slope on AL** | -0.007 (0.006) | -0.006 (0.008) | -0.046 (0.042) | -0.066 (0.087) | 962.72 (0.07) | 509 (1) | .80 | .046 | .066 |
| *N* = 859. E3/E3 group *n* = 597. E3/E4 group *n* = 262. All χ^2^_SB_ *p*s < .001. All estimates are standardized. _SB_ = Satorra-Bentler. *RMSEA* = root mean square error of approximation. *SRMR* = standardized root mean square residual. | | | | | | | | | |
