# Supplementary material for: Apolipoprotein E genotype does not moderate the associations of depressive symptoms, neuroticism and allostatic load with cognitive ability and cognitive aging in the Lothian Birth Cohort 1936
Source: PLoS One. 2018 Feb 16;13(2):e0192604. doi: 10.1371/journal.pone.0192604 (PMC5815580; doi:10.1371/journal.pone.0192604)
Supplement: S4 Appendix — Main and moderation effect test results, including for mediation models, in models where years of education was added as a predictor of the general cognitive ability intercept and slope. (DOCX) [file pone.0192604.s004.docx]

**Results from models with education added as a covariate**

Tables N-P contain the main, moderation and mediation effect results for models with years of education added as a predictor of the general cognitive ability intercept and slope. The average years of education were similar across the groups of *APOE* E4 non-carriers (*M* [*SD*] = 10.74 [1.10], *Mdn* = 10, *range* = 8–13, *skew* = 0.79, *kurtosis* = -0.41) and carriers (*M* [*SD*] = 10.74 [1.19], *Mdn* = 10, *range* = 7–14, *skew* = 0.71, *kurtosis* = -0.16).

Overall, the results from these models were similar to the results from models without education as a covariate. Most notably, the effects of depressive symptoms, neuroticism and AL on baseline cognitive ability tended to be attenuated by around 20-30% (maximum Δ = 0.07). Consequently, the effect of AL on baseline cognitive ability only remained statistically significant in the model in which this regression was jointly estimated across groups. Also, in the non-E4 carriers group, there were large attenuations for the regressions of cognitive change on depressive symptoms (maximum Δ = 0.39) and AL (maximum Δ = 0.41), while in the E4 carriers group, the standard errors tended to be much larger for all effects on cognitive change. This meant that with one exception, these effects were no longer statistically significant. In the mediation models, estimates for the regression of AL on neuroticism were very similar, but this path did become statistically significant in the E4 non-carriers group in each model.

Crucially, like in models without education as a covariate, *APOE* E4 status did not moderate the effects of depressive symptoms, neuroticism and AL on baseline cognitive ability and subsequent cognitive decline. Plus, importantly, AL did not mediate the effects of neuroticism on cognitive ability and decline.

| **Table N. Main Effect and Moderation Tests in Models with Education Added as a Covariate.** | | | | | | | | | | |
| --- | --- | --- | --- | --- | --- | --- | --- | --- | --- | --- |
| **Predictor** | **Regression constrained** | **Regression of intercept on predictor *estimate* (*SE*)** | | **Regression of slope on predictor *estimate* (*SE*)** | | **χ^2^_SB_ (Δχ^2^_SB_)** | ***df* (Δχ^2^_SB_ *df*)** | **Δχ^2^_SB_ *p*** | ***RMSEA*** | ***SRMR*** |
|  |  | **no E4 group** | **E4 group** | **no E4 group** | **E4 group** |  |  |  |  |  |
| **Depressive symptoms** | **None** | -0.13  (0.04)*** | -0.18  (0.06)** | -0.55  (0.28)* | -0.54  (1.45) | 835.48 (N/A) | 406 (N/A) | N/A | .045 | .063 |
| **Depressive symptoms** | **Intercept** | -0.14  (0.03)*** | -0.15  (0.04)*** | -0.54  (0.28) | -0.67  (1.16) | 835.73 (0.25) | 407 (1) | .62 | .045 | .063 |
| **Depressive symptoms** | **Slope** | -0.13  (0.04)*** | -0.17  (0.06)** | -0.49  (0.27) | -0.85  (0.51) | 836.15 (0.35) | 407 (1) | .55 | .045 | .063 |
| **Neuroticism** | **None** | -0.18  (0.04)*** | -0.23  (0.07)*** | -0.39  (0.34) | -0.05  (2.46) | 968.37 (N/A) | 502 | N/A | .043 | .061 |
| **Neuroticism** | **Intercept** | -0.19  (0.03)*** | -0.22  (0.04)*** | -0.37  (0.34) | -0.14  (2.35) | 968.41 (0.04) | 503 (1) | .85 | .042 | .061 |
| **Neuroticism** | **Slope** | -0.19  (0.04)*** | -0.22  (0.06)*** | -0.30  (0.34) | -0.68  (0.77) | 968.08 (0.18) | 503 (1) | .67 | .042 | .061 |
| **Allostatic load** | **None** | -0.08  (0.04) | -0.07  (0.06) | -0.43  (0.33) | -0.85  (0.56) | 844.79 (N/A) | 406 (N/A) | N/A | .046 | .065 |
| **Allostatic load** | **Intercept** | -0.07  (0.03)* | -0.08  (0.04)* | -0.43  (0.33) | -0.84  (0.59) | 844.79 (0.02) | 407 (1) | .90 | .046 | .065 |
| **Allostatic load** | **Slope** | -0.08  (0.04) | -0.07  (0.06) | -0.44  (0.30) | -0.82  (0.52) | 844.15 (0.00) | 407 (1) | .95 | .046 | .065 |
| *N* = 1028. Non-E4 carriers group *n* = 722. E4 carriers group *n* = 306. All χ^2^_SB_ *p*s < .001. All estimates are standardized. _SB_ = Satorra-Bentler. *RMSEA* = root mean square error of approximation. *SRMR* = standardized root mean square residual.  **p* ≤ .05. ***p* ≤ .01. ****p* ≤ .001. | | | | | | | | | | |

| **Table O*.* Parameter Estimates from Mediation Models with Education Added as a Covariate.** | | | | | | | | | | |
| --- | --- | --- | --- | --- | --- | --- | --- | --- | --- | --- |
| **Regression constrained to equality** | **Intercept on N**  ***est.* (*SE*)** | | **Slope on N *est.* (*SE*)** | | **AL on N**  ***est.* (*SE*)** | | **Intercept on AL *est.* (*SE*)** | | **Slope on AL**  ***est.* (*SE*)** | |
|  | **no E4 group** | **E4 group** | **no E4 group** | **E4 group** | **no E4 group** | **E4 group** | **no E4 group** | **E4 group** | **no E4 group** | **E4 group** |
| **None** | -0.18  (0.04)*** | -0.23  (0.07)*** | -0.32  (0.31) | -0.10  (1.28) | 0.08  (0.04)* | 0.08  (0.07) | -0.06  (0.04) | -0.05  (0.06) | -0.39  (0.30) | -0.81  (0.54) |
| **Intercept on AL** | -0.18  (0.04)*** | -0.22  (0.07)*** | -0.32  (0.30) | -0.01  (1.35) | 0.08  (0.04)* | 0.08  (0.07) | -0.06  (0.03) | -0.07  (0.04) | -0.40  (0.29) | -0.79  (0.60) |
| **Slope on AL** | -0.18  (0.04)*** | -0.22  (0.07)*** | -0.32  (0.30) | -0.00  (1.41) | 0.08  (0.04)* | 0.08  (0.07) | -0.06  (0.04) | -0.06  (0.06) | -0.40  (0.27) | -0.78  (0.52) |
| *N* = 1028. Non-E4 carriers group *n* = 722. E4 carriers group *n* = 306. All estimates are standardized. N = neuroticism. AL = allostatic load.  **p* ≤ .05. ***p* ≤ .01. ****p* ≤ .001. | | | | | | | | | | |

| **Table P*.* Tests of Mediation and Moderated Mediation in Models with Education Added as a Covariate.** | | | | | | | | | |
| --- | --- | --- | --- | --- | --- | --- | --- | --- | --- |
| **Regression constrained to equality** | **Indirect effect on intercept *est.* (*SE*)** | | **Indirect effect on slope *est.* (*SE*)** | | **χ^2^_SB_ (Δχ^2^_SB_)** | ***df* (Δχ^2^_SB_ *df*)** | **Δχ^2^_SB_ *p*** | ***RMSEA*** | ***SRMR*** |
|  | **no E4 group** | **E4 group** | **no E4 group** | **E4 group** |  |  |  |  |  |
| **None** | -0.005 (0.004) | -0.004 (0.006) | -0.032 (0.031) | -0.063 (0.073) | 1016.32 (N/A) | 540 (N/A) | N/A | .041 | .059 |
| **Intercept on AL** | -0.005 (0.004) | -0.005 (0.005) | -0.033 (0.031) | -0.061 (0.074) | 1016.29 (0.05) | 541 (1) | .83 | .041 | .059 |
| **Slope on AL** | -0.005 (0.004) | -0.004 (0.006) | -0.033 (0.029) | -0.061 (0.068) | 1015.82 (0.01) | 541 (1) | .94 | .041 | .059 |
| *N* = 1028. Non-E4 carriers group *n* = 722. E4 carriers group *n* = 306. All *n*s for E4 carriers group = 306. All χ^2^_SB_ *p*s < .001. All estimates are standardized. _SB_ = Satorra-Bentler. *RMSEA* = root mean square error of approximation. *SRMR* = standardized root mean square residual. | | | | | | | | | |
